# Disrupting *fzd9b* in Zebrafish Recapitulates Stress- and Anxiety-Like Behaviours Relevant to Williams Syndrome

**DOI:** 10.1007/s12035-026-06127-w

**Published:** 2026-08-24

**Authors:** Jose V. Torres-Perez, Adele Leggieri, Xian Wang, Aine Kehoe, Lianri Van Schalkwyk, Aleksandra M. Mech, Sofia Anagianni, William Havelange, Caroline H. Brennan

**Affiliations:** 1 Departament de Biologia Cel·lular, Biologia Funcional i Antropologia Física, Universitat de València, 46100 Burjassot, València Spain; 2 School of Biological and Behavioural Sciences, Queen Mary University of London, London, E1 4NS UK; 3 Department of Basic and Clinical Neuroscience, Maurice Wohl Clinical Neuroscience Institute, Institute of Psychiatry, Psychology and Neuroscience, King’s College London, London, UK

**Keywords:** Williams-Beuren syndrome, Wnt-signalling, GSK, BIO-acetoxime

## Abstract

Williams syndrome (WS) is a multifaceted developmental disorder characterized by a spectrum of physical and intellectual traits. Individuals with WS exhibit friendly, impulsive, and hyper-social behaviours, often coupled with anxiety. WS is caused by a hemizygous microdeletion on chromosome 7q11.23. Among the affected genes, *FZD9* is implicated in neural development and synaptic function. Thus, we postulated that disruption of *FZD9* might contribute to the behavioural features of WS, and that pharmacological modulation of Wnt signalling, particularly the canonical β-catenin pathway, could offer therapeutic potential. We generated two zebrafish lines carrying predicted loss-of-function alleles of *fzd9b*, a zebrafish orthologue of *FZD9*. Our results demonstrated that mutant fish exhibited altered expression of hypothalamic–pituitary–interrenal axis-related genes, increased neuronal branching, impaired habituation to acoustic startle, and increased anxiety-like behaviour in adulthood. Canonical Wnt/β-catenin pathway gene expression was also altered, together with the expression of other frizzled receptors; however, acute GSK-3 inhibition failed to rescue behavioural phenotypes under the conditions tested. These findings indicate that the selected acute treatment paradigm was insufficient to reverse the phenotype but do not exclude a contribution of canonical Wnt signalling. Expression of *fzd9b*, *wnt5b*, and *tafa5l* was upregulated, further supporting transcriptional dysregulation. Together, our results highlight a conserved role for Fzd9b in modulating stress reactivity and anxiety-like behaviour in zebrafish and support FZD9 as a contributor to WS-associated neurobehavioural alterations.

**Supplementary Information:**

The online version contains supplementary material available at 10.1007/s12035-026-06127-w.

## Background

Williams syndrome (WS, or Williams-Beuren syndrome) is a developmental disorder involving intellectual disability alongside distinct physical and behavioural traits [1, 2]. Physical features may include growth delay, heart problems, facial alterations, hypodontia, and short stature. Behaviourally, WS is marked by hypersociability, impulsivity, and reduced attention span. Despite a strong drive to socialise, individuals with WS often show anxiety responses that appear to conflict with their sociability [3], the nature of which remains elusive.

Although individuals with WS tend to be highly sociable, anxiety (especially in social settings) and related disorders are highly prevalent, representing a leading mental health concern in this population [2, 4]. Anxiety symptoms are suggested to worsen with age [4, 5]. Importantly, anxiety responsiveness in WS appears linked to alterations in the hypothalamic–pituitary–adrenal (HPA) axis, which may impair regulation of emotional reactivity, particularly in socially demanding situations [6–8].

WS is caused by a hemizygous microdeletion on chromosome 7q11.23, typically occurring *de novo* and affecting males and females equally [9, 10]. This deletion spans approximately 25–28 genes, including several with established developmental roles [11], making it challenging to link individual genes to specific WS-associated endophenotypes. Nonetheless, studies of individuals with atypical deletions, where certain genes are retained, have revealed genotype–phenotype correlations [12, 13]. Using iPSC-derived neurons from neurotypical and WS individuals, including one with a preserved *FZD9* gene (pWS88), Chailangkarn et al. [12] found that neurons from WS cases with the full deletion exhibited increased dendritic length, number, and spine density. In contrast, neurons from the pWS88 case resembled controls [12] suggesting that *FZD9* deficiency might be responsible for the WS-associated neuronal abnormalities.

*FZD9* encodes frizzled 9, a transmembrane receptor of the frizzled family involved in Wnt signalling [14]. FZD9 binds to WNT proteins and has been linked to dendritic spine formation [15] and axonal growth [16]. Mice lacking functional *Fzd9* display learning and memory impairments [17]. During neural tube development, *Fzd9* expression is first detected in neural precursor cells and remains elevated in the brain, heart, testis and skeletal muscle from development into adulthood [14, 17, 18].

Disrupted Wnt signalling is linked to psychiatric and neurological disorders including bipolar disorders and schizophrenia, where anxiety is highly prevalent [19, 20]. Dysregulation of the Wnt pathway has also been associated with stress-related behaviours in depression [21] and attention-deficit hyperactivity disorders [22]. Wnt signalling modulates stress responses via β-catenin expression in the nucleus accumbens and hippocampus [23, 24]. Inhibition of GSK3β, a key player in the canonical Wnt signalling pathway, produces anxiolytic and pro-social effects [25], and Wnt pathway modulation with sulindac or LiCl influences behavioural responses and recovery in stress models. Potentially relevant for therapeutics, Korem et al. demonstrated in a murine model of shock and extinction that modulating the canonical Wnt/β-catenin pathway can influence behavioural responses to stress and facilitate recovery [23].

Taking all together, we hypothesised that disrupted FZD9 signalling contributes to anxiety-related phenotypes in WS, and that restoring Wnt signalling pharmacologically may offer therapeutic benefit. To test our hypothesis, we used zebrafish (*Danio rerio*), which share conserved neuro-endocrinal circuitry [26] and high genetic homology with other vertebrates [27]. As recent findings suggest *baz1b*, another of the WS-affected genes, may be more directly linked to WS social traits [28], we further hypothesised that disruption of *fzd9b* would have limited effects on social behaviour. We generated zebrafish lines carrying predicted loss-of-function alleles of *fzd9b* and assessed both social and anxiety-related behaviours. Upon identifying anxiety-related alterations, we tested whether acute treatment with 6-bromoindirubin-3'-oxime (BIO-acetoxime), a selective GSK-3 inhibitor [29], could revert the stress-related phenotype.

## Results

### CRISPR/Cas9-Mediated Disruption of *fzd9b* in Zebrafish

**Fig. 1:**
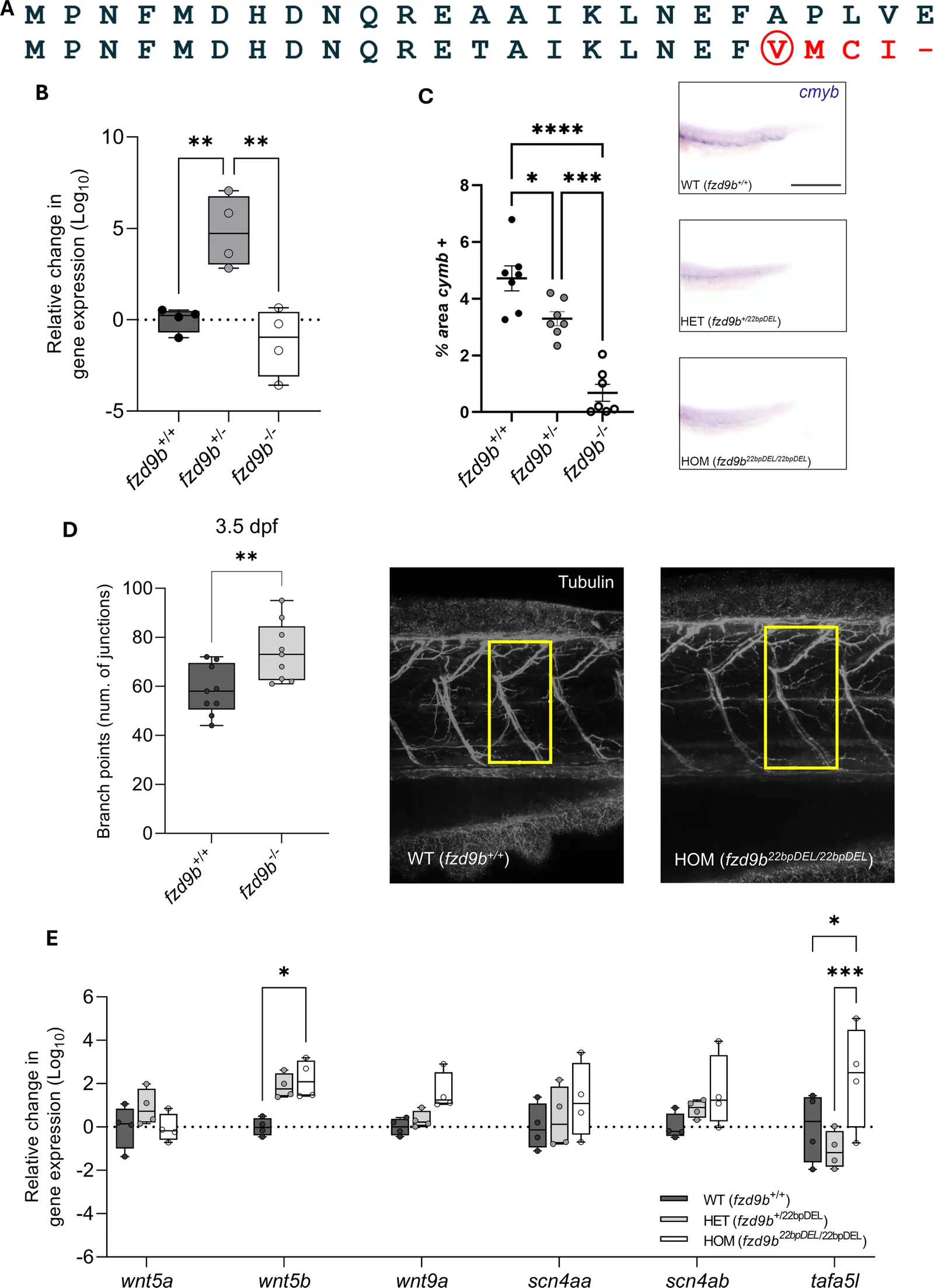
Characterization of the *fzd9b* 22 bp deletion (*fzd9b*^*22bpDEL*^) line. **A**) BLAST alignment of the predicted amino acids (aa) sequences for wildtype Fzd9b (WT, top) and the products predicted from the mutant allele (bottom), showing the region of exon 1 used for genotyping (see Sup. Fig. 2). Red circle marks the mutation starting site, altered residues are in red and stop codon is shown as a dash (-). **B**) Relative gene expression (log_10_ scale) of *fzd9b* in 5 dpf larvae; each point represents a pool of 16 larvae (*N* = 4 per genotype); bars show mean ± standard error mean (SEM). **C**) Quantification of the whole mount *in situ* hybridization (ISH) for *cmyb* at the tail of 40 hpf embryos (*N* = 7 per genotype); right panel shows representative images. **D**) Quantification of neuronal branch points following acetylated tubulin immunostaining and skeleton analysis in larval tails; graphs show individual values and mean ± SEM; representative images are shown alongside with yellow rectangles depicting areas analysed. **E)** qPCR analysis of *wnt5a*, *wnt5b*, *wnt9a*, *scn4aa*, *scn4ab* and *tafa5l* expression in the same 5 dpf larval pools used in panel B; graphs show box-and-whiskers (min to max) with mean as a central line; dots represent biological replicates. Statistical details are provided in Sup. Table 1. In all cases: * *p* < 0.05, ** *p* < 0.01, *** *p* < 0.001, **** *p* < 0.000 vs. corresponding pairwise comparison; Scale bars represent 25 µm

Two zebrafish lines carrying predicted loss-of-function (LoF) alleles of *fzd9b* were generated using CRISPR/Cas9: *fzd9b*^*22bpDEL*^ and *fzd9b*^*52bpDEL*^, carrying a 22 and a 52-base pair (bp) deletion respectively (Fig. 1, and Sup. Fig. 1−2). Both deletions lead to a frameshift and the introduction of a premature stop codon (Fig. 1A, Sup. Fig. 1A). Quantitative PCR (qPCR) using primers located upstream of the mutation site revealed a significant (ANOVA *fzd9b*^*22bpDEL*^: p = 0.0011, Mean increase = 4.793; ANOVA *fzd9b*^*52bpDEL*^: p = 0.0103, Mean increase = 3.755) upregulation of *fzd9b* mRNA expression in heterozygous (*fzd9b*^+/-^) animals compared to wild type (*fzd9b*^+*/*+^, WT) (*fzd9b*^*22bpDEL*^: *fzd9b*^+/-^ vs. *fzd9b*^+*/*+^ p = 0.0021; *fzd9b*^*52bpDEL*^: *fzd9b*^+/-^ vs. *fzd9b*^+*/*+^ p = 0.0033, Fig. 1B, Sup. Fig. 1B).

As *fzd9b* is a single-exon gene, and nonsense-mediated decay typically requires splicing to recognise aberrant transcripts [30], these findings suggest that both full-length and smaller truncated Fzd9b proteins (3D structure in Sup. Fig. 3) may be expressed. Additional qPCR using primers downstream of the mutation site confirmed a similar expression pattern (data not shown).

In zebrafish, Fzd9b is known to affect haematopoietic stem cells (HPSC) development [31]. To obtain functional evidence that the generated alleles impair Fzd9b activity, and to assess downstream effects on haematopoiesis, we performed in situ hybridization (ISH) experiments in 40 h post fertilization (hpf) embryos using a riboprobe targeting *cmyb*, a marker of HPSC expansion [31]. Both mutant lines showed significantly reduced *cmyb* expression compared to *fzd9b*^+*/*+^ siblings: *fzd9b*^*22bpDEL*^ (*fzd9b*^+*/*+^ vs *fzd9b*^+/-^ p = 0.0219,*fzd9b*^+*/*+^ vs *fzd9b*^*−/−*^ *p* < 0.0001, *fzd9b*^+/-^ vs *fzd9b*^*−/−*^ p = 0.0001,Fig. 1C) and *fzd9b*^*52bpDEL*^ (*fzd9b*^+*/*+^ vs *fzd9b*^+/-^ p = 0.0050; *fzd9b*^+*/*+^ vs *fzd9b*^*−/−*^ *p* < 0.0001, *fzd9b*^+/-^ vs *fzd9b*^*−/−*^ *p* < 0.0001; Sup. Fig. 1C).

To assess whether *fzd9b* disruption was associated with altered neuronal morphology during development, as reported in cultured and postmortem neurons [12], we performed whole-mount immunostaining against acetylated tubulin and quantified neuronal branch points in the tail following skeletonization analysis. In the *fzd9b*^*22bpDEL*^ line, *fzd9b*^*−/−*^ mutants (M = 74.00, SD = 12.03) showed a significant increase in the number of branch points compared to WT siblings (M = 58.44, SD = 10.06,t(16) = 2.975, *p* < 0.05; Fig. 1D), whereas no significant differences were detected between *fzd9b*^+*/*+^ and *fzd9b*^+/-^ larvae (*fzd9b*^+*/*+^: M = 21.58, SD = 5.418; *fzd9b*^+/-^: M = 18.67, SD = 5.657; t(19) = 1.198, p = 0.2455). In contrast, no significant differences were detected in the *fzd9b*^*52bpDEL*^ line in either comparison performed (*fzd9b*^+*/*+^ vs *fzd9b*^+/-^: *fzd9b*^+*/*+^: M = 21.58, SD = 5.418; *fzd9b*^+/-^: M = 26.67, SD = 6.555; t(22) = 2.071, p = 0.0503; *fzd9b*^+*/*+^ vs *fzd9b*^*−/−*^: *fzd9b*^+*/*+^: M = 58.44, SD = 10.06; *fzd9b*^*−/−*^: M = 54.78, SD = 13.60; t(16) = 0.6502, p = 0.52485; Sup. Fig. 1D).

Due to the stronger and more consistent phenotypes observed in the *fzd9b*^*22bpDEL*^ line (see further results), this model was prioritised for detailed characterisation, while the *fzd9b*^*52bpDEL*^ line was examined in supplementary analyses to assess mutation-specific variability (see Supplementary Material).

### *fzd9b* Disruption Alters Expression of Downstream Genes at Larval Stages

To investigate downstream molecular effects of *fzd9b* disruption, we performed qPCR experiments targeting genes known or predicted to interact with Fzd9b (Fig. 1E): *wnt5a/b*, as WNT5A requires FZD9 to enhance dendritic spine density in rodents [15],*wnt9a*, known to interact with Fzd9b in zebrafish [31],and *scn4aa/b* and *tafa5l* (syn. *FAM19A5*), the expression of which is altered in neural progenitor cells and neurons, respectively, from classical WS patients but not from pWS88 individuals [12].

We observed that *wnt5b* was significantly upregulated in *fzd9b*^*−/−*^ when compared to *fzd9b*^+*/*+^ siblings (p = 0.0241; Fig. 1E), as well as *tafa5l* (*fzd9b*^*−/−*^ vs. *fzd9b*^+*/*+^: p = 0.0159; *fzd9b*^*−/−*^ vs. *fzd9b*^+/-^: p = 0.0003; Fig. 1E). No other post-hoc comparisons reached significance for this line (p > 0.05; Fig. 1E; Sup. Table 1). Furthermore, although not reaching significance, *fzd9b*^+/-^ showed a tendency for increased *wnt5b* expression levels (p = 0.0663; Fig. 1E).

### Disruption of *fzd9b* Does Not Alter Responses to Light–Dark Transitions at Larval Stages

To assess stress-related behaviour, we performed the forced light–dark transition (FLDT) assay in 5 days post-fertilization (dpf) zebrafish larvae. The FLDT paradigm measures the locomotor response to sudden changes in environmental illumination and has previously been used to evaluate stress reactivity [32–35].

There was a main effect of time in basal locomotion (baseline), light and dark periods with no effect of genotype and no genotype by time interaction (Sup. Fig. 4A and Sup. Table 2 for *p*-values). In response to the transition from dark to light, fish displayed a typical startle and freezing response, characterised by reduced movement, followed by a gradual recovery to baseline activity over the 10-min light phase. Locomotor changes during this period, assessed as the cumulative slope of activity during the light phases, also showed no significant genotype effect (Sup. Table 2).

### Disruption of *fzd9b* Alters Habituation to Acoustic Startle at Larval Stages

Habituation to repeated acoustic startle is commonly used to assess associative learning [33, 36, 37]. However, the magnitude of the startle response and the rate of habituation can also serve as indicators of hyperarousal and exaggerated stress reactivity [38, 39]. In this assay, isolated zebrafish larvae were exposed to repeated acoustic cues while their movement was recorded as a readout of stress and habituation. A brief flash of light was presented at the beginning and end of the protocol to assess stimulus specificity.

Time had a significant main effect on distance travelled during both baseline (prior to the first flash of light and before the first acoustic stimulus (χ2 (8) = 24.0650, *p* < 0.05)) and the tap events (χ2 (19) = 658.2177, *p* < 0.0001). However, we did not observe a significant effect of genotype (*p* > 0.05).

**Fig. 2:**
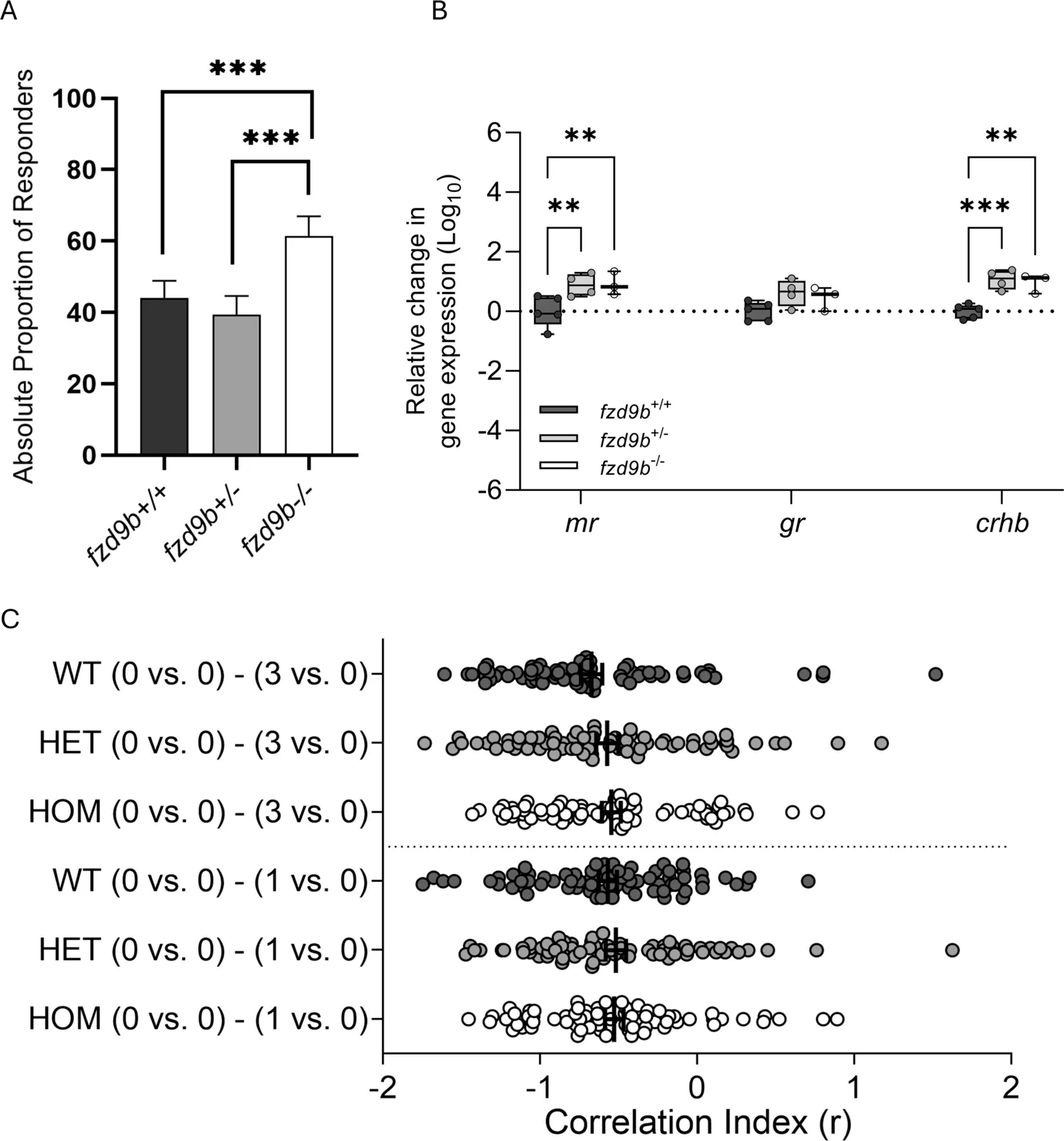
Disrupting *fzd9b* leads to delayed habituation to acoustic startle and altered expression of HPI-axis-related genes. **A**) Proportion of responders across all acoustic stimuli in 5 dpf larvae: for *fzd9b*^+*/*+^ (*N* = 45), *fzd9b*^+/-^ (*N* = 91), and *fzd9b*^*−/−*^ (*N* = 43); data show mean ± SEM. **B**) Relative expression (log_10_ scale) of HPI axis genes (*mr, gr* and *crhb*) measured by qPCR in pooled 5 dpf larvae (*N* = 5–3 per genotype, 16 larvae per pool); graphs show individual values and mean ± SEM. **C**) Social preferences correlation index (r) in juvenile zebrafish (20–22 dpf) for all contrasts tested (0 vs. 0, vs. 0 vs. 1 or vs. 0 vs. 3) in *fzd9b*^+*/*+^ *(*dark grey), *fzd9b*^+/-^ (light grey) or *fzd9b*^*−/−*^ (white); each point represents an individual; bars show mean ± SEM. In all cases: ** *p* < 0.01, *** *p* < 0.001, **** *p* < 0.000 vs. corresponding pairwise comparison

When assessing the proportion of responders to the startle stimuli (Fig. 2A), *fzd9b*^+*/*+^ larvae showed a habituation response to repeated acoustic startle consistent with previous reports [33, 40]: 91% of *fzd9b*^+*/*+^ zebrafish responded to the first acoustic stimulus, but only 25% of *fzd9b*^+*/*+^ responded to the last. When considering all acoustic stimuli together, there was a significant main effect of genotype on the overall proportion of responders (χ2 (2) = 56.126, *p* < 0.0001), as well as a significant two-way stimulus events by genotype interaction (χ2 (22) = 1.411411, *p* < 0.0001) such that *fzd9b*^*−/−*^ habituated significantly more slowly than *fzd9b*^+*/*+^ (*p* < 0.0001) and *fzd9b*^+/-^ (*p* < 0.0001). During the flash of light stimuli (before and after the tap events) there were no significant differences between genotypes in either the magnitude of response or the slope of recovery (p > 0.05).

### Disruption of *fzd9b* Alters the Expression of HPI-Axis-Related Genes at Larval Stages

To further characterise the anxiety-related phenotype associated with *fzd9b* disruption, we assessed by qPCR the expression of genes related to the hypothalamic-pituitary-interrenal (HPI) axis, the equivalent to the mammalian HPA axis: mineral corticoid receptor (*mr*), glucocorticoid receptor (*gr*) and corticotropin releasing hormone b (*crhb*).

There was a significant upregulation (ANOVA: *p* < 0.001) of *mr* in both *fzd9b*^+/-^ (p = 0.0051) and *fzd9b*^*−/−*^ (p = 0.0076), and of *crh* in both *fzd9b*^+/-^ (p = 0.008) and *fzd9b*^*−/−*^ (p = 0.0049) when compared to *fzd9b*^+*/*+^ siblings (Fig. 2B). No other comparisons reached significance (p > 0.05).

### Disrupting *fzd9b* Does Not Affect Sociability at 3 Weeks of Age

Increased sociability is a hallmark of WS and may interfere with the expression of anxiety-related behaviours [1, 2, 4, 6–8]. To assess whether *fzd9b* participates in zebrafish social abilities, we performed a ‘sociability assay’ at 3 weeks of age, when social preference is fully established [41]. Zebrafish were placed in a U-shaped arena with clear compartments at each end, one containing either one or three conspecifics, and preference for social zones was measured.

All zebrafish showed a significant preference for both one (*fzd9b*^+*/*+^: −2368, *fzd9b*^+/-^: −2150, *fzd9b*^*−/−*^: −2192; *p* < 0.001;) and three (*fzd9b*^+*/*+^: −2246, *fzd9b*^+/-^: −2130, *fzd9b*^*−/−*^: −2207; *p* < 0.001) conspecifics.

Additionally, to evaluate whether social drive varied by genotype, we also compared correlation indexes (r) as previously described [28]. We found no significant differences between genotypes (H (6) = 432, p = 0.2562,Fig. 2C).

### *fzd9b* Mutants Show Altered Anxiety-Like Behaviour at Adult Stages

**Fig. 3:**
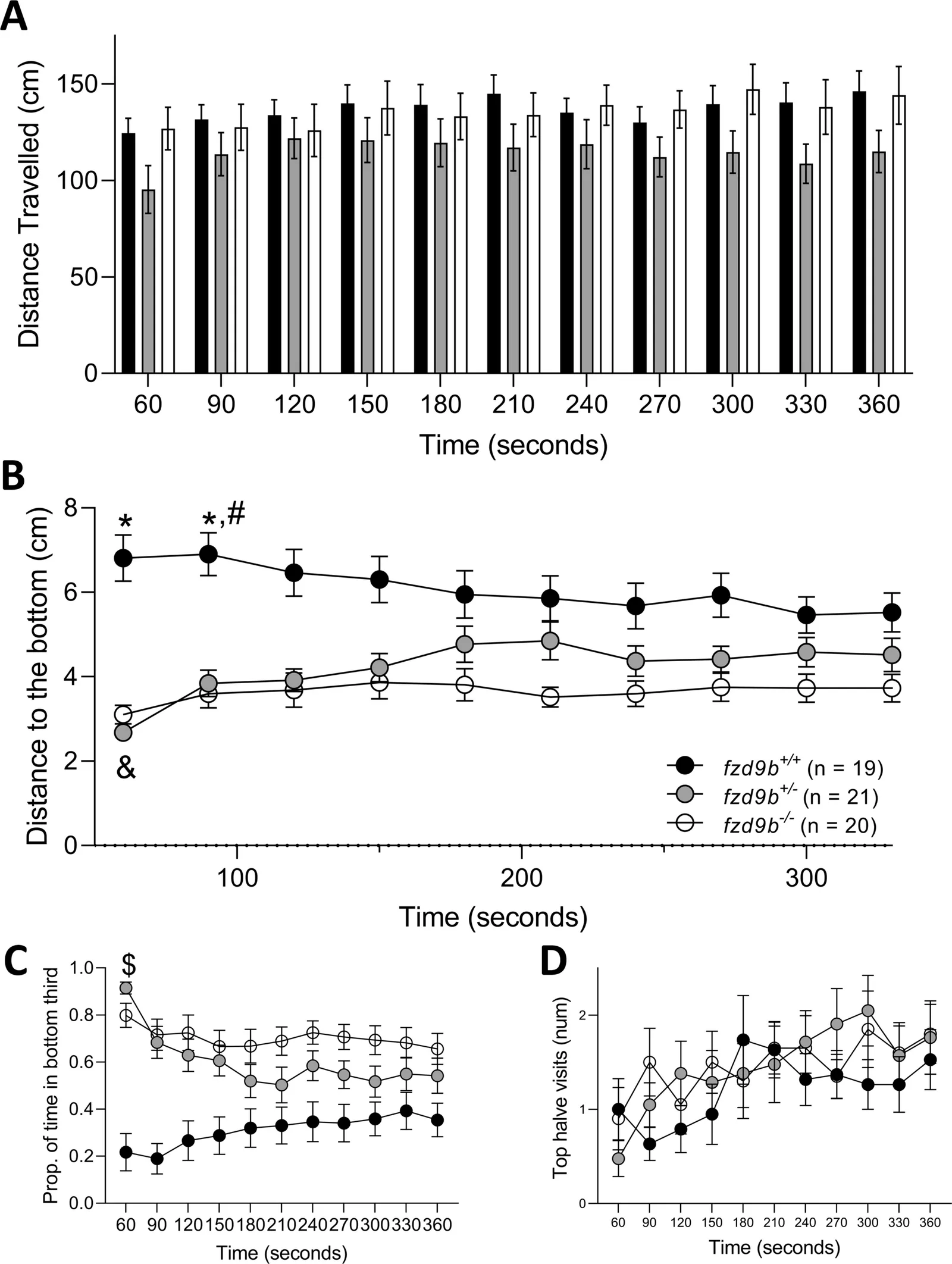
Novel tank diving assay reveals altered anxiety-like behaviour in *fzd9b* mutants. **A**) Total distance travelled by adult fish from the three genotypes (*fzd9b*^+*/*+^, *fzd9b*^+/-^, *fzd9b*^*−/−*^) over time in the assay, shown in 30-s time bins. **B**) Distance to the tank bottom over time for each genotype; sample sizes (n) are indicated in the panel. **C**) Proportion of time spent in the bottom third of the tank. **D**) Number of visits to the top half of the tank. Graphs show mean ± SEM. * *p* < 0.0 5 vs. 9th time point from same genotype (WT); # *p* < 0.0 5 vs. 10th time point from same genotype (WT); # *p* < 0.0 5 vs. 4th time point until the last, from same genotype (HET); & *p* < 0.0 5 vs. 4th time point until the last, from same genotype (HET); $ *p* < 0.0 5 vs. 5th time point until 7th and from 9th until the last, from same genotype (HET)

Anxiety is a common feature of WS thought to exacerbate with age [2, 4, 5]. To assess whether *fzd9b* disruption affected anxiety-like behaviour in adult zebrafish, we used the novel tank diving assay (Fig. 3), in which time spent near the bottom and distance from the bottom serve as established indicators of anxiety-like responses [42].

We observed a significant effect of time on total distance travelled (F (10) = 26.4103, p = 0.003), with locomotion increasing after the first-time bin (1st time bin vs. 4th-7th, 9th, 11th: *p* < 0.05; Fig. 3A). We observed no other significant effect (p > 0.05).

The analysis of average distance from the bottom revealed a significant main effect of genotype (F (2) = 27.103, *p* < 0.001; Fig. 3B) driven by *fzd9b*^+*/*+^ fish having a significantly greater distance from the bottom than *fzd9b*^+/-^ and *fzd9b*^*−/−*^ (*fzd9b*^+*/*+^ vs. *fzd9b*^+/-^: *p* < 0.001; *fzd9b*^+*/*+^ vs. *fzd9b*^*−/−*^: *p* < 0.001). Time did not have a main effect (F (10) = 15.923, p = 0.1019), but there was a significant time-by-genotype interaction (F (20) = 97.432, *p* < 0.001). Over the course of the assay, *fzd9b*^+*/*+^ fish gradually reduced their distance from the bottom over time (1st vs. 9th time bin: p = 0.05; 2nd vs. 9th: p = 0.018; vs. 10th: p = 0.033) while *fzd9b*^+/-^ increased their distance (1st vs. 4th: p 0.002; vs. 5th-11th: *p* < 0.001) and *fzd9b*^*−/−*^ did not show any significant trend.

The analysis of time spent on the bottom third (Fig. 3C) also differed significantly by genotype (F (2) = 213.4849, *p* < 0.001). All genotypes differed significantly (*p* < 0.001): *fzd9b*^+*/*+^ spent the least time in the bottom third, *fzd9b*^*−/−*^ the most, and *fzd9b*^+/-^ showed an intermediate pattern distinct from both. There was no main effect of time (F (10) = 4.5732, p = 0.9178), but a significant interaction between time by genotype interaction (F (20) = 39.2124, *p* < 0.001). Post-hoc tests revealed that only *fzd9b*^+/-^ fish showed a change over time, spending significantly less time in the bottom third in later bins (1st time bin vs. 5th-7th: *p* < 0.05; vs. 9th-11th: *p* < 0.05).

Finally, we assessed the top half visits (Fig. 3D). We observed a significant effect of time (F (10) = 39.1785, *p* < 0.001), whereby fish increased top half visits over time (1st time bin vs. 4th-11th: *p* < 0.05), consistent with a reduced anxiety-like phenotype as trial progressed. We observed no other significant effect (p > 0.05).

### Disruption of *fzd9b* Alters Transcriptional Targets of the Wnt/Β-Catenin Signalling Pathway

To explore whether the behavioural phenotypes observed in *fzd9b* mutants were associated with dysregulation of canonical Wnt signalling, we performed qPCR analyses of established Wnt/β-catenin pathway targets in 5 dpf larvae. Genes included in the panel were *cyclind1* and *myca*, which are classical downstream targets of β-catenin [43–45],*ctnnb1* and *ctnnb2*, encoding β-catenin isoforms [46], *axin2*, a negative feedback regulator of the pathway [43, 47],and *sfrp2*, an antagonist of Wnt signalling [48, 49].

**Fig. 4:**
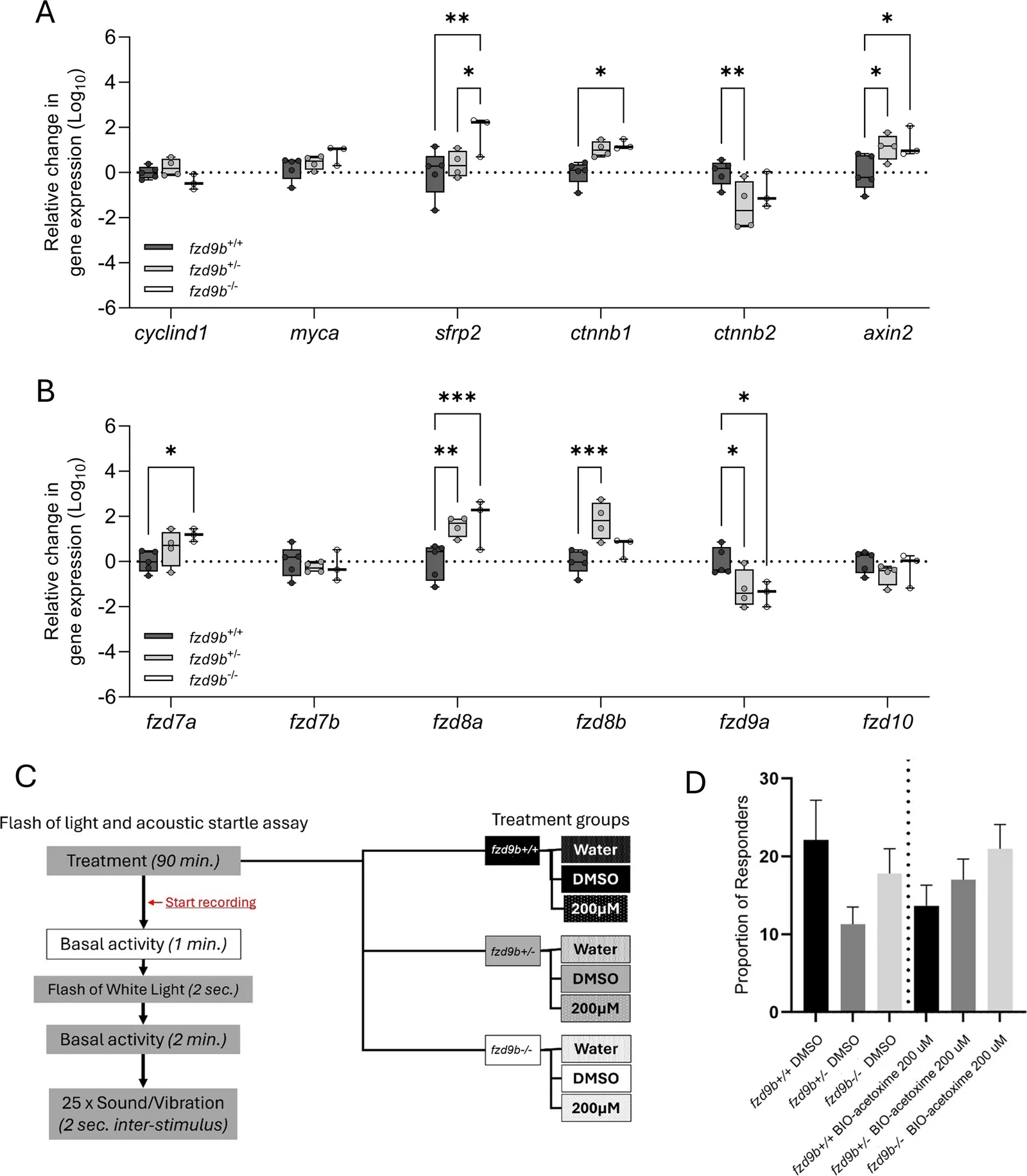
Wnt pathway expression and behavioural response to BIO-acetoxime in 5 dpf zebrafish. **A**) Relative gene expression (log₁₀ scale) of Wnt/β-catenin pathway genes (*cyclind1*, *myca*, *ctnnb1*, *ctnnb2*, *axin2*, *sfrp2*) measured by qPCR in pooled 5 dpf larvae (*n* = 3–5 per genotype; 16 larvae per pool). Graphs show individual values and mean ± SEM. **B**) Relative gene expression (log₁₀ scale) of other frizzled receptor genes (*fzd7a*, *fzd7b*, *fzd8a*, *fzd8b*, *fzd9a*, and *fzd10*) measured by qPCR (*n* = 3 per genotype; 16 larvae per pool). Graphs show individual values and mean ± SEM. **C**) Schematic of the drug treatment and behavioural assay. After 90 min of incubation with BIO-acetoxime (200 μM) in darkness, zebrafish were recorded for 1 min (baseline), exposed to 2 s of white light, followed by 2 min darkness, and then subjected to 25 consecutive acoustic stimuli (2-s inter-stimulus interval). Treatment groups included: *fzd9b*^+*/*+^ (control: water; vehicle: DMSO; drug: BIO-acetoxime), *fzd9b*^+/-^ and *fzd9b*^*−/−*^ (each treated with water, DMSO, or BIO-acetoxime). **D)** Proportion of responders across stimuli for each treatment condition; each genotype × treatment group included *n* = 42 larvae; data represent mean ± SEM. * *p* < 0.05 vs. corresponding pairwise comparison

We observed a significant upregulation of *sfrp2* and *axin2* expression in both *fzd9b*^+/-^ and *fzd9b*^*−/−*^ larvae when compared to *fzd9b*^+*/*+^ counterparts (Fig. 4A; Sup. Table 1). In addition, *ctnnb1* expression was significantly increased in *fzd9b*^*−/−*^ larvae, whereas *ctnnb2* was significantly increased in *fzd9b*^+/-^ larvae (Fig. 4A; Sup. Table 1). Together, these findings indicate that disruption of *fzd9b* is associated with broader alterations in Wnt/β-catenin-related transcription.

### Disruption of *fzd9b* Alters the Expression of Other Frizzled Receptors

To determine whether disruption of *fzd9b* affected the expression of other members of the frizzled receptor family, we performed qPCR analysis of those previously reported relevant for neural development: *fzd7a*, *fzd7b*, *fzd8a*, *fzd8b*, *fzd9a* and *fzd10* (Fig. 4B; Sup. Table 1) [31]. We observed a significant increase in *fzd7a* expression in *fzd9b*^*−/−*^ larvae, whereas *fzd7b* and *fzd10* expression were not significantly altered. *fzd8a* expression was significantly increased in both *fzd9b*^+/-^ and *fzd9b*^*−/−*^ larvae, while *fzd8b* was significantly increased in *fzd9b*^+/-^ larvae only. In contrast, *fzd9a* expression was significantly reduced in both mutant genotypes compared to WT siblings. These data indicate that disruption of *fzd9b* is associated with selective transcriptional changes in other frizzled receptors, including altered expression of the closely related paralogue *fzd9a*.

### Acute GSK-3 Inhibition is Insufficient to Rescue Altered Stress-Reactivity in *fzd9b* Mutant Larvae

To test whether acute pharmacological modulation of the canonical Wnt/β-catenin pathway could reverse the behavioural alterations observed in *fzd9b* mutants, we treated larvae with BIO-acetoxime, a selective GSK-3 inhibitor, at 200 µM, the highest tolerated concentration that does not affect basal locomotion in zebrafish larvae [29]. GSK-3 plays a central role in the canonical Wnt/β-catenin pathway by promoting β-catenin degradation in the absence of pathway activation [50]. Interestingly, inhibition of GSK3 might have a therapeutic potential in different neurological disorders and psychiatric diseases [50].

As we saw no genotype differences in larval FLDT assay and cost precluded adult behavioural testing, we used the repeated acoustic startle as a measure of stress-reactivity. Thus, after 90 min of drug-exposure, zebrafish were subjected to the acoustic startle assay (Fig. 4C). To account for potential impact of the drug on vision, we included a flash of light, as with prior acoustic startle assay, to determine whether potential differences in response were due to fatigue.

Consistent with previous findings, no genotype differences were observed in basal locomotion before the flash of light or before the acoustic stimuli (Sup. Table 3). Likewise, we found no differences across genotypes in the magnitude of response to the flash of light, nor in the recovery slope. However, during the tap events following DMSO exposure, a significant main effect of genotype and a significant time-by-genotype for distance travelled: *fzd9b*^*−/−*^ larvae travelled significantly more than *fzd9b*^+*/*+^ siblings (*p* < 0.05). While the absolute proportion of responders did not differ between genotypes (p > 0.05), the rate of habituation over time showed a significant main effect of genotype and a significant genotype-by-stimulus interaction. Specifically, *fzd9b*^*−/−*^ larvae habituated significantly more slowly than both *fzd9b*^+*/*+^ and *fzd9b*^+/-^ (*p* < 0.0001 for both), with no difference between the latter two (p > 0.05).

Treatment with BIO-acetoxime had no significant effect on basal locomotion or responses to the light flash (Sup. Table 3). During the acoustic startles, no significant effect of genotype on distance travelled was found (p > 0.05). However, a significant three-way interaction (genotype × drug × time) was detected, indicating that *fzd9b*^*−/−*^ larvae treated with BIO-acetoxime travelled greater distance than both BIO-acetoxime treated *fzd9b*^+*/*+^ and *fzd9b*^+/-^ counterparts (*p* < 0.05).

Regarding the proportion of responders, BIO-acetoxime had no effect on the absolute response rates (p > 0.05; Fig. 4D). However, there was a significant main effect of genotype on the rate of habituation such that *fzd9b*^*−/−*^ larvae showed slower habituation (Sup. Table 3). Additionally, there was a main effect of treatment, where BIO-acetoxime generally reduced habituation efficiency (*p* < 0.0001), along with a significant genotype-by-drug interaction and a three-way interaction (Sup. Table 3). Post-hoc comparisons confirmed that *fzd9b*^*−/−*^ larvae treated with BIO-acetoxime habituated significantly more slowly than treated *fzd9b*^+*/*+^ and *fzd9b*^+/-^ (*p* < 0.0001 for both).

### Consistent Findings Across the *fzd9b*^*52bpDEL*^ Line and the Primary Mutant Line

Although the *fzd9b*^*52bpDEL*^ line was not the primary focus, supplementary analyses revealed broadly similar disruptions to those seen in the *fzd9b*^*22bpDEL*^ line. These included altered habituation to acoustic startle (Sup. Fig. 5A), changes in the expression of HPI-axis-related genes (Sup. Fig. 5B), mild sociability differences in *fzd9b*^+/-^ fish (Sup. Fig. 5C), and reduced anxiety-like behaviour in adults (Sup. Fig. 6). In contrast to the broader transcriptional changes observed in the main line, the supplementary line showed more limited alterations in Wnt/β-catenin-related and frizzled receptor gene expression (Sup. Fig. 7A-B). BIO-acetoxime treatment likewise did not rescue the behavioural phenotype under the acute treatment conditions tested in this line (Sup. Fig. 7C). Differences between the lines may reflect variation in the predicted truncated products or genetic background. A detailed comparison is provided in Table 1.

**Table 1:** Comparison of molecular and behavioural phenotypes in the two zebrafish line carrying predicted *fzd9b* LoF alleles

Phenotype/Marker	*fzd9b*^*22bpDEL*^ (Main Line)	*fzd9b*^*52bpDEL*^ (Supplementary)
↑ *fzd9b* mRNA (in *fzd9b*^+/-^)	✓	✓
↓ *cmyb* expression (ISH)	✓ (in *fzd9b*^+/-^ and *fzd9b*^*−/−*^)	✓ (in *fzd9b*^+/-^ and *fzd9b*^*−/−*^)
↑ neuronal branching	✓ (in *fzd9b*^*−/−*^)	✗ (no differences)
↑ *wnt5b* expression	✓ (in *fzd9b*^*−/−*^)	✓ (in *fzd9b*^+/-^)
↑ *tafa5l* expression	✓ (in *fzd9b*^*−/−*^)	✓ (in *fzd9b*^+/-^)
Forced light–dark transitions (FLDT)	✗ (no differences)	✗ (no differences)
Acoustic startle: slower habituation	✓ (in *fzd9b*^*−/−*^)	✓ (in *fzd9b*^+/-^)
Altered expression of HPI-axis-related genes (*mr*, *gr*, *crhb*)	✓ (*crh* ↑ and *mr* ↑ in *fzd9b*^+/-^ and *fzd9b*^*−/−*^)	✓ (*crh* ↑ in *fzd9b*^+/-^)
Sociability (3 wpf)	✗	✓ (↑ in *fzd9b*^+/-^ only in 0 vs. 3)
Novel tank diving (anxiety-like behaviour)	✓ (↑ anxiety in *fzd9b*^+/-^ and *fzd9b*^*−/−*^)	✓ (↓ anxiety in *fzd9b*^+/-^ only)
↑ Wnt pathway target genes	✓ (*sfrp2*↑ and *axin2*↑ in *fzd9b*^*−/−*^ and *fzd9b*^*−/−*^; *ctnnb1*↑ in *fzd9b*^*−/−*^, and *ctnnb2*↑ in *fzd9b*^+/-^)	✓ (*cyclind1*↑ and *sfrp2*↑ in *fzd9b*^+/-^)
*frizzled* receptors	✓ (*fzd7a*↑ in *fzd9b*^*−/−*^, *fzd8b*↑ in *fzd9b*^+/-^, *fzd8a*↑ and *fzd9a*↓ in *fzd9b*^+/-^ and *fzd9b*^*−/−*^)	✓ (*fzd8b*↑ and *fzd9a*↑ in *fzd9b*^+/-^)
Rescue following acute BIO-acetoxime treatment (GSK-3 inhibitor)	✗ (no effect under conditions tested)	✗ (no effect under conditions tested)

Arrows indicate direction of change (↑ = increased; ↓ = decreased); ticks (✓) indicate significant effects and (✗) indicates no significant change.

## Discussion

We generated a zebrafish line carrying a predicted loss-of-function allele of *fzd9b* (*fzd9b*^*22bpDEL*^) to test whether disrupted Fzd9b signalling underlies altered stress reactivity, anxiety and social behaviour, traits associated with WS. Mutant larvae showed impaired habituation to acoustic startle alongside altered expression of HPI-axis-related genes, consistent with altered stress regulation. Adults also exhibited anxiety-like behaviours. However, acute pharmacological treatment targeting the Wnt/β-catenin pathway failed to reverse these phenotypes under the conditions tested. This finding does not exclude a contribution of canonical Wnt signalling, as the lack of rescue may reflect developmental timing, irreversible circuit alterations established before treatment, insufficient or temporally limited pathway activation, or the combined involvement of canonical and non-canonical mechanisms. The observed increase in *wnt5b* expression nevertheless suggests that non-canonical Wnt-related mechanisms may contribute these behavioural phenotypes. A second line (*fzd9b*^*52bpDEL*^, presented as supplementary validation) replicated several key findings.

### Disrupting *fzd9b* Alters the Expression of *fzd9b*, *wnt5b* and *tafa5l* at Larval Stages and is Associated with Altered Neuronal Branching

In zebrafish, *fzd9b* is a single exon gene. Consequently, transcripts carrying premature termination codons may escape conventional exon-junction-dependent nonsense-mediated decay, which typically relies on exon-exon junctions to recognise aberrant mRNAs [45]. The mutant transcripts may therefore give rise to truncated Fzd9b proteins lacking substantial portions of the receptor, including intracellular regions required for normal receptor activation and downstream signalling [51]. However, because protein expression was not assessed directly, the production, stability, localisation and functional properties of these predicted truncated products remain unknown. Although the generated alleles are predicted to markedly impair Fzd9b function, residual or altered activity cannot be excluded.

Interestingly, *fzd9b*^+/-^ fish showed a significant increase in *fzd9b* mRNA levels, whereas *fzd9b*^*−/−*^ subjects did not. This may reflect a genotype-dependent transcriptional response to disruption of *fzd9b* rather than a linear gene-dosage effect. Distinct transcriptional responses between heterozygous and homozygous mutants have also been reported in other zebrafish CRISPR models, consistent with non-linear gene dosage effects [52]. Importantly, the expression of *cmyb*, a marker of haematopoietic stem cell expansion positively regulated by Fzd9b [31], was reduced in both *fzd9b*^+/-^ and *fzd9b*^*−/−*^ mutants, with the greatest reduction in *fzd9b*^*−/−*^. These data provide functional support that the lines generated here disrupt Fzd9b activity, consistent with previous studies using morpholino or CRISPR/Cas9 approaches to knockdown *fzd9b* in haematopoietic endothelium [31]. Nevertheless, these findings do not establish whether the alleles produce a complete absence of Fzd9b function.

In addition, acetylated tubulin immunostaining revealed increased neuronal branch points in homozygous mutants. Although this analysis was performed in tail neurons and does not directly assess dendritic spine density in the brain, it nonetheless supports the idea that disruption of *fzd9b* affects early neuronal morphology *in vivo*. This finding is broadly consistent with previous evidence linking FZD9 to neuronal structural development and with reports of altered neuronal morphology in WS-related cellular models [12]. Importantly, this result further supports the selection of the *fzd9b*^*22bpDEL*^ line as the primary line for detailed characterisation. However, further work will be required to define the specific brain regions and cell types in which *fzd9b* contributes to these phenotypes *in vivo*.

The expression of *wnt5b*, a ligand for Fzd9b, but not of *wnt5a*, was upregulated in the *fzd9b*^*−/−*^ fish. In rodents, WNT5a and FZD9 interact to mediate an increase in density of dendritic spines [15]. The increase in *wnt5b* expression may reflect a broader transcriptional adjustment in Wnt-related signalling following disruption of *fzd9b*, although this remains to be tested directly. Our data suggest that in zebrafish, contrary to rodents, Wnt5b rather than Wnt5a may participate in related signalling processes, although we did not establish a direct interaction between Wnt5b and Fzd9b. Interestingly, the number and size of dendritic spines is significantly increased in WS individuals. Hence, altered Wnt-related transcription, potentially involving ligands such as Wnt5b acting through other Frizzled receptors, could contribute to the neuronal alterations associated with WS [12]. The absence of significant differences in the supplementary *fzd9b*^*52bpDEL*^ line suggests that this phenotype may be sensitive to allele-specific effects or broader genotype-dependent regulatory responses.

A similar upregulation of *tafa5l* (also named *fam19A5*), previously reported to be altered in WS patient-derived neurons [12], was observed in the *fzd9b*^*−/−*^ fish. Thus, this finding suggests that, similar to humans, the expression of *tafa5l* may also be influenced by disruption of Fzd9b-related signalling in zebrafish. *tafa5l* encodes a chemokine-like protein involved in cell proliferation and migration via G protein-coupled receptors. Expression of this protein appears to be largely restricted to brain structures [53] and has been associated with cognitive impairment in vascular dementia [54].

In the supplementary *fzd9b*^*52bpDEL*^ line, related but not identical transcriptional changes were observed, which may reflect allele-specific effects arising from variation in the predicted truncated products, differences in transcript or protein stability, or residual background variation. Interestingly, the predicted truncated protein generated by the 52-bp deletion contains a stretch of mismatched amino acids that includes three cysteine residues. This structure may enhance its affinity for cysteine-rich domains, such as that of Sfrp2, a secreted Wnt antagonist known to modulate Wnt signalling by binding to both Wnt ligands and Frizzled receptors [51, 55]. Thus, it is plausible that this Fzd9b truncated protein generated in the 52-bp deletion line could interfere with the normal Wnt pathway dynamics by sequestering Sfrp2 or altering its function, potentially contributing to the behavioural phenotype observed in heterozygotes. Furthermore, the upregulation of canonical Wnt targets observed in heterozygous fish from the 52-bp deletion line (not present in homozygotes for the same line, nor in any mutant fish from the 22-bp deletion line) suggests genotype- and line-specific modulation of Wnt activity, possibly driven by structural differences in the truncated peptides. While the precise mechanisms remain unclear, these data point to a complex interplay between Fzd9b, Wnt signalling, and Sfrp2. Overall, these findings point to a complex relationship between *fzd9b* disruption and Wnt-related transcription that may differ between alleles.

### *fzd9b* Mutants Display Altered Stress and Anxiety-Like Behaviours, but Limited Impact on Sociability

To assess the behavioural consequences of *fzd9b* disruption, we first evaluated stress responses at larval stages. Stress reactivity was assessed using the FLDT paradigm and the acoustic startle response. FLDT assay is an established test to assess differences in stress-reactivity in zebrafish [32–35]. In wildtype 5 dpf larvae, transitions from dark to light trigger a freezing response, while light to dark transitions induce a locomotor spike, both followed by recovery. The magnitude and recovery rate of these responses reflect stress reactivity or anxiety-like levels [33]. Mutants showed no differences in the light–dark transition, suggesting that forced changes in environmental light did not affect their stress reactivity.

Nonetheless, *fzd9b*^*−/−*^ fish displayed delayed habituation to repeated acoustic startle, suggesting hyper-arousal. The repeated acoustic startle assay is primarily used to study startle and habituation responses to sudden sound stimuli, which can be associated with alterations in the fish's stress response [28, 34]. Although the startle response is a complex behaviour influenced by various factors, a longer time to habituate (or lack of habituation) indicates increased sensitivity to stressors. In addition, our HPI axis gene expression analysis, including increased *crhb* expression in both mutant genotypes, is consistent with a transcriptional signature of altered stress regulation that may contribute to the behavioural phenotype.

These findings align with a similar response to the acoustic startle assay in a mouse model for WS, where deletion of the WS critical region led to increased startle reactivity, reflecting heightened sensory sensitivity and autonomic hyper-responsiveness associated with the syndrome [56]. Interestingly, hypersensitivity to sound (hyperacusis) is a common feature in WS [57], and increased auditory startle reflex, associated with autonomic hyper-reactivity, is a prevalent characteristic in children with anxiety disorders [58].

Adult mutant zebrafish exhibited increased anxiety-like behaviour in the novel tank diving assay, spending more time near the tank bottom compared to wildtypes. In this assay, the anxiety-like response is measured as the time and distance fish spent near the bottom of the tank [42]. Both *fzd9b*^+/-^ and *fzd9b*^*−/−*^ spent overall more time close to the bottom, which is a sign of an increased anxious-like phenotype. This suggests that Fzd9b plays a role in modulating anxiety-like responses throughout development.

In contrast, mutant zebrafish did not show significant changes in sociability at three weeks of age in the main line of this study. Although Wnt signalling has been associated with pro-social behaviours in rodents [25], our results indicate that *fzd9b* loss does not have a robust or consistent effect on sociability and is therefore unlikely to be a major driver of the hypersociability observed in WS. While *fzd9b* disruption clearly impacts stress- and anxiety-related phenotypes, its role in modulating social behaviour appears limited. In this context, recent work by our group and others has highlighted *BAZ1B*, another gene commonly missing in WS, as a key regulator of sociability via its influence on neural crest maturation [28, 59, 60].

Findings from the supplementary line, *fzd9b*^*52bpDEL*^, broadly aligned with the main line, with mutants showing altered habituation to acoustic startle and changes in the expression of HPI-axis-related genes, consistent with altered stress regulation. However, unlike the main line, *fzd9b*^+/-^ fish from this line also displayed a mild increase in sociability and reduced anxiety-like behaviour in adulthood, consistent with greater top-half exploration. These line-specific differences reinforce our view that any effect of *fzd9b* on sociability is subtle and not robust across alleles. This may reflect the interplay between sociability and anxiety reported in WS [4], although *fzd9b* appears to be a minor contributor. Similar behavioural variability between mutant lines has been reported following early-life alterations in HPI-axis regulation in zebrafish [61].

The differences in phenotypic severity between the two lines may reflect allele-specific variation in the expression, stability or residual activity of the predicted truncated Fzd9b products. Differences in genetic background or allele-dependent transcriptional responses may also contribute, as distinct mutant alleles can elicit different molecular and phenotypic outcomes in zebrafish [62, 63]. However, the underlying mechanisms remain unknown, and the shared alterations in stress-related behaviour across both lines support a common effect of *fzd9b* disruption.

### Canonical Wnt-Related Transcription is Altered in *fzd9b* Mutants, but Acute GSK-3 Inhibition is Insufficient to Rescue the Behavioural Phenotype

Previous studies have linked Wnt signalling, particularly the canonical Wnt/β-catenin pathway, to the modulation of anxiety-related behaviours [23, 25]. In our study, the main *fzd9b* mutant line showed altered expression of several Wnt/β-catenin-related genes, including increased expression of *sfrp2* and *axin2* in both mutant genotypes, alongside genotype-specific increases in *ctnnb1* and *ctnnb2*. These findings indicate that disruptions of *fzd9b* is associated with broader alterations in canonical Wnt-related transcription [48, 49]. In addition, we observed selective changes in the expression of other frizzled receptors, including increased *fzd7a*, *fzd8a* and *fzd8b* expression, together with reduced *fzd9a* expression. Although we cannot completely exclude indirect effects related to the mutagenesis strategy, the observed reduction in *fzd9a* expression is more likely to reflect altered transcriptional regulation within the frizzled receptor network than direct disruption of the *fzd9a* locus. This interpretation is supported by the fact that *fzd9a* and *fzd9b* were quantified using gene-specific primers and that the mutation was generated and genotyped at the *fzd9b* locus. These findings suggest that disruption of *fzd9b* may trigger broader transcriptional adjustments within the frizzled receptor family, and that *fzd9a* and other paralogues may influence the molecular response to *fzd9b* disruption, potentially through transcriptional adaptation or other allele-dependent regulatory responses. However, Sfrp2 has also been reported to act as a Wnt agonist in certain cellular contexts via the non-canonical pathway [64]. Similarly, *wnt5b*, a non-canonical Wnt ligand [15], was upregulated in mutant fish, suggesting that the phenotype observed in our study might also involve alterations in the non-canonical pathway.

To explore whether canonical Wnt signalling mediates the stress-related phenotypes, we exposed larvae to BIO-acetoxime, a GSK-3 inhibitor that activates β-catenin signalling [29]. Acute treatment did not rescue the altered habituation phenotype under the conditions tested. However, because pathway activation was not confirmed molecularly, these findings do not conclusively establish whether canonical Wnt signalling contributes to the phenotype. The lack of rescue could reflect insufficient or temporally limited pathway activation, treatment after relevant developmental or circuit-level alterations had already been established, or the combined contribution of canonical and non-canonical Wnt signalling. Given the short interval between treatment and behavioural testing, transcriptional changes in canonical Wnt target genes may also not have been detectable within the experimental window.

A similar lack of behavioural rescue was observed in the supplementary line, despite only more limited transcriptional changes being detected, namely increased *cyclind1* and *sfrp2* in heterozygous fish. These findings suggest that, although disruption of *fzd9b* can alter canonical Wnt-related transcription, the precise molecular signature varies between mutant alleles and that the acute GSK-3 inhibition paradigm used here was insufficient to produce behavioural rescue. Although we observed selective changes in the expression of other *frizzled* receptors, the present study does not establish whether these transcriptional changes result in functional compensation following *fzd9b* disruption. Addressing this question will require spatial expression analyses and combinatorial genetic approaches. Together, these data support a complex contribution of Wnt signalling to the phenotype, potentially involving both canonical and non-canonical components, rather than a mechanism that can be reversed by short-term GSK-3 inhibition alone.

## Conclusion and Final Remarks

Disruption of *fzd9b* in zebrafish resulted in consistent alterations in molecular markers and stress- and anxiety-driven behaviours, highlighting its potential contribution to WS endophenotypes. These effects were not rescued by acute GSK-3 inhibition under the conditions tested; although this finding does not exclude a contribution of canonical Wnt signalling, it indicates that the selected acute treatment paradigm was insufficient to reverse the behavioural phenotype. Importantly, zebrafish carrying the predicted *fzd9b* LoF alleles did not display alterations in sociability, suggesting a dissociation between anxiety and social phenotypes within the WS-associated genomic region. This complements our previous findings on *baz1b* [28], and highlights the need to establish gene-specific characterizations in multigenic syndromes such as WS.

Our study is not without limitations. The subtle differences observed between the two mutant lines may reflect allele-specific differences in the expression, stability or residual activity of the predicted truncated proteins, or residual background variation. Because Fzd9b protein expression was not assessed directly, we cannot confirm whether these truncated products are produced or determine their biological properties. Moreover, although we observed selective changes in the expression of other frizzled receptors, we did not assess their spatial expression patterns or test their functional contribution; therefore, we cannot conclude whether these changes reflect transcriptional adaptation or have any functional compensatory effect. In addition, we did not directly map *fzd9b* expression in specific brain regions or cell types, and therefore the precise anatomical substrates underlying the behavioural phenotypes remain to be determined. Likewise, although we detected altered expression of HPI-axis-related genes, these transcriptional changes to not establish functional dysregulation of the HPI axis; our interpretation of potential HPI axis dysregulation relies on indirect molecular readouts, including altered expression of *mr* and *crhb*. Direct endocrine measurements, such as basal or post-stress cortisol levels, were not performed and will be required to determine whether HPI-axis activity is altered. Although this approach is commonly used in the field, it remains a limitation of the present study. Similarly, although BIO-acetoxime is a selective GSK-3 inhibitor and GSK-3 inhibition has previously been used to modulate canonical Wnt signalling in zebrafish, pathway engagement was not confirmed molecularly under the present experimental conditions. Consequently, the absence of behavioural rescue cannot distinguish between insufficient or temporally limited pathway activation, developmental or circuit-level changes that were no longer reversible at the time of treatment, or a phenotype involving both canonical and non-canonical Wnt signalling.

In addition, adult behavioural testing was performed using fish from mixed-sex tanks, but sex was not recorded for individual animals at the time of testing; therefore, sex-specific effects could not be assessed. Moreover, although the novel tank assay revealed clear genotype-dependent differences in bottom dwelling, the relatively flat exploratory profile of the control group may limit the broader generalisability of this behavioural readout. Finally, while extensive outcrossing was used to minimise genetic confounds, off-target or linked mutations cannot be entirely ruled out.

Future studies should aim to dissect the relative contributions of canonical and non-canonical Wnt pathways to Fzd9b's influence on stress responses and to explore therapeutic strategies targeting these circuits. In addition, given the multigenic nature of WS, future work should examine potential gene–gene interactions between *fzd9b* and other WS-associated genes, including their possible combined contribution to social phenotypes. Furthermore, although our study proposes that *fzd9b* contributes to the stress- and anxiety-alterations associated while having little or no impact on their social abilities, an interplay between anxiety and social functioning (driven by *FZD9*-related genetic networks or independently) might still persist in WS [65, 66]. Future investigations attempting to assess the combined anxiety and social phenotypes might elucidate the existence of synergies.

## Materials and Methods

### Animal Husbandry

Zebrafish were from a Tübingen background and maintained at ~ 25–28 °C in a recirculating water system under a 14:10 light–dark cycle. Fish were fed twice daily with dry food (ZM-400, Zebrafish Management Ltd) or live *Artemia salina*. Breeding and rearing followed established protocols [28]. To follow the 3Rs and reduce costs, 5 dpf behavioural assays without drug treatment used offspring from heterozygous in-crosses (*fzd9b*^+/-^ x *fzd9b*^+/-^), allowing for genotype blinding. All other experiments used offspring from ≥ 5 breeding pairs of defined genotypes: wild-type offspring from *fzd9b*^+*/*+^ x *fzd9b*^+*/*+^ crosses, homozygous offspring from *fzd9b*^*−/−*^ x *fzd9b*^*−/−*^ crosses, and heterozygous offspring from *fzd9b*^+*/*+^ x *fzd9b*^*−/−*^ crosses. Adult breeders had previously been genotyped by fin clipping and were maintained in genotype-defined tanks. Breeding occurred over 2–3 consecutive days per trial, with different fish each day. Parental contributions were balanced by sex and genotype where relevant. Eggs were pooled by genotype (if known), and to avoid tank effects, at least five Petri dishes containing 30–50 fertilised eggs per genotype were reared separately each day.

### Generation of Zebrafish Carrying Predicted *fzd9b* Loss-of-Function (LoF) Alleles

We used a similar CRISPR/Cas9 strategy to that described by Keatinge and colleagues [67] to generate predicted LoF alleles. Nonetheless, we implemented similar small variations as previously reported in our group [28]. Although zebrafish have two paralogues for *fzd9* (*fzd9a* and *fzd9b*, Sup. Fig. 8), previous expression analyses suggest that *fzd9b* is the more relevant paralogue in the developmental neural contexts examined here. Specifically, *fzd9b* is expressed in the dorsal neural tube at stages relevant to neural crest-derived melanocyte specification, whereas *fzd9a* was reported to show a broader/ubiquitous expression pattern at earlier stages and no specific expression in early melanoblasts at 30 hpf [68]. Although this does not exclude a contribution of *fzd9a* to other aspects of neural development or function, these expression data provided the rationale for targeting *fzd9b* in the present study (Sup. Fig. 8) [68]. Therefore, we tailored our CRISPR/Cas9 strategy to the zebrafish gene *fzd9b* (Ensembl gene ID: ENSDARG00000014673).

The crRNA (5’ACAGCCAUACUCCACCAGAG) was designed to overlap a restriction site for Bsl1 including a PAM site and located within the upstream (5’) half portion of this single exon gene. Primers used for genotyping: forward 5’AGGGCATCGGATACAACCTC and reverse 5’TTCAAAGATTCAGGCCAGGC (Sup. Fig. 2). Once a pair of founders was identified, they were in-crossed and resulting F1 genotyped. All identified *fzd9b*^*−/−*^ individuals from the F1 were individually outcrossed with different non-related wildtype fish, eggs collected and combined to randomly select a population of (at least two tanks of 50 individuals each) *fzd9b*^+/-^ zebrafish to constitute the F2. F2s were in-crossed and F3 genotyped to establish a breeding stock of fish from each genotype. Experimental analysis started from generation F5 onwards, always obtained by multiple outcrosses followed by genotyping. Zebrafish lines can be requested from the corresponding authors.

### Quantitative PCR (qPCR)

Whole-body larvae at 5 dpf and at visually similar developmental stages were collected in pools of 16 individuals per sample. Therefore, each biological replicate consisted of pooled RNA from 16 whole larvae of the same genotype (*N* = 3–5 biological replicates per genotype, obtained from different clutches). Larval genotype was determined by the parental cross: *fzd9b*^+*/*+^  × *fzd9b*^+*/*+^ crosses generated wild-type larvae, *fzd9b*^*−/−*^ × *fzd9b*^*−/−*^ crosses generated homozygous larvae, and *fzd9b*^+*/*+^  × *fzd9b*^*−/−*^ crosses generated heterozygous larvae. Individual larvae were therefore not biopsied or genotyped before RNA extraction. Samples were stored in RNAlater (Thermo Fisher) until use. Total RNA was extracted and reverse transcribed into cDNA using standard procedures as previously described [28]. Primer sequences, target gene information, and additional details are provided in Supplementary Table 4.

Quantitative PCR was performed to assess expression of *fzd9b*, downstream candidate genes, HPI axis genes, canonical Wnt signalling targets, and other frizzled genes. Primers for *fzd9b* were designed upstream of the mutation site to detect both wild-type and mutant transcripts. Expression of *fzd9b* and general profiling of downstream targets were quantified using SYBR Green-based qPCR, as described in [28]. To evaluate stress-related signalling, expression of HPI axis genes (*mr*, *gr*, and *crhb*) was measured from the same RNA samples. To investigate potential modulation of Wnt signalling and whether loss of *fzd9b* altered the expression of other frizzled receptors relevant to neural development [31], separate sets of qPCRs were performed using newly obtained RNA from similarly staged larvae. For these experiments, cDNA was synthesised using the LunaScript RT SuperMix Kit (E3010, NEB Ltd., UK), and qPCR was performed using the Luna® Universal qPCR Master Mix Kit on a Bio-Rad CFX96 Touch Real-Time PCR Detection System.

Gene expression was normalised to two reference genes in all experiments and relative expression levels were analysed using the modified Pfaffl method reported previously [28]. The amplification efficiencies of both the genes of interest and the reference genes were incorporated into the analysis. Expression of *fzd9b* and the initial downstream target panels was normalised to *ef1a* and *rpl13a*, whereas expression of HPI-axis genes, canonical Wnt pathway targets, and other members of the *frizzled* receptor family was normalised to *ef1a* and *actb2*. These experiments were performed at different times, and the reference-gene pairs were selected according to the primer sets available in the laboratory at the time of each experiment.

### Whole Body *in situ* Hybridization (ISH) and % of Area Stained

Zebrafish embryos from all genotypes (*N* = 7 per group, from different clutches) were collected at 40 hpf, carefully staged to exclude developmental differences, and fixed overnight in 4% paraformaldehyde (PFA; Sigma, Gillingham, UK) in phosphate-buffered saline (PBS). The following day, embryos were washed in PBS with 0.05% Tween-20, dehydrated through a graded methanol series (25–100%, 5 min per step), and stored in 100% methanol at –20 °C until use.

ISH was performed as previously described [28]. Briefly, antisense RNA probes against *cmyb* (ENSDART00000075726.5) were generated by PCR from cDNA using the following primers: Forward 5′-CTGCTAAAGTCAGCCCAACTCC and Reverse 5′-ATCGTCTGCTCTTCCGTCTTCC. Stained embryos were imaged laterally using a Leica MZ75 stereomicroscope.

Quantification of the stained area was performed in Fiji [69]. Images were rotated to align tails horizontally, converted to 8-bit greyscale, equalised, and binarized using Phansalkar’s method [70]. A rectangular region of interest (ROI) spanning from the end of the yolk sac to the end of the yolk sac extension was defined to include the full tail. The percentage of positive staining within the ROI was calculated and used for analysis.

### Acetylated Tubulin Immunostaining and Analysis of Neuronal Branching

To assess neuronal morphology in *fzd9b* mutant larvae, whole-mount immunostaining for acetylated tubulin was performed at larval stages (*n* = 9–12). Immunostaining was performed broadly as previously described [71]. Comparisons were conducted separately for *fzd9b*^+*/*+^ versus *fzd9b*^+/-^ larvae at 2.5 dpf and *fzd9b*^+*/*+^ versus *fzd9b*^*−/−*^ larvae at 3.5 dpf. Stained larvae were imaged from the side using a confocal microscope (20 × objective) with identical laser settings across samples within each experiment, and 35 μm z-stacks were acquired in the Alexa Fluor 546 channel. Images were taken from the tail region.

Maximum projections were generated in Fiji, and neuronal branching was quantified using the Skeleton plugin. Briefly, equal-sized rectangular ROIs were drawn over the selected tail region, and the number of junctions was determined for the third, fourth and fifth neurons from the end of the tail bud. These values were used as a readout of neuronal branch points for statistical analysis.

### Behavioural Experimental Design

#### Forced Light–Dark Transition (FLDT, 5 dpf)

Stress-related behaviour was assessed at 5 dpf using the FLDT paradigm [28, 72]. Larvae were singly housed and recorded under infrared light for a 10-min baseline period, followed by three cycles of alternating 10-min light and dark phases. Locomotor activity was recorded using EthoVision XT and exported in 1-s and 1-min bins. Tests were performed in parallel between 09:00 and 16:00 over three consecutive days, with two independent experiments per day. Offspring of *fzd9b*^+/-^  × *fzd9b*^+/-^ crosses were used to ensure the experimenter remained blind to genotype, genotyping was performed after testing (as mentioned above).

#### Flash of Light and Repeated Acoustic Startle (5 dpf)

This assay was used to assess stress reactivity and habituation. At 5 dpf, larvae were individually placed in 24-well plates under dark conditions. Following a 10-min baseline, fish were exposed to a 2-s light flash, followed by 1-min recovery. Next, they received 20 consecutive acoustic/vibration stimuli (2-s interstimulus interval), followed by an additional 2-s light flash. Testing occurred between 09:00 and 16:00. Distance travelled was recorded and analysed as in the FLDT assay.

#### Social Preference Assay (3 Weeks Post-Fertilisation)

Social behaviour was assessed in juvenile zebrafish (20–22 dpf) using the assay developed by Dreosti and colleagues [41], with modifications as previously described [28]. Fish were tested between 10:00 and 19:00, matched with stimulus fish of similar genotype, age, and size. Tests were run in parallel using two DanioVision Observation Chambers, with balanced stimulus presentation sides and at least two independent experiments conducted daily. Position and motility were tracked with EthoVision XT (Noldus), with data outputted in 15-min bins. Social Preference Index (SPI) and Correlation Index (r = SPI_ExperimentalPhase_ – SPI_AcclimationPeriod_) were calculated as previously described [28].

#### Novel Tank Diving Assay (3 Months)

Anxiety-like behaviour in adults was assessed using the novel tank diving assay [42]. To minimise potential tank effects, fish from at least two independent tanks per genotype (*fzd9b*^+*/*+^, *fzd9b*^+/-^ and *fzd9b*^*−/−*^) were used. Fish were taken from mixed-sex tanks containing both males and females,however, sex was not recorded for individual animals at the time of behavioural testing and was therefore not included as a factor in the statistical analysis. Tests were conducted between 09:00 and 14:00 over two consecutive days. Individual fish were placed in a novel trapezoidal tank containing fresh system water (replaced every 2–3 trials) and recorded from the side for 6 min. The initial segment corresponding to fish placement in the tank was excluded from the analysis, resulting in an analysed interval of approximately 5 min. Distance from the bottom and total motility were tracked and analysed using EthoVision XT, with data binned into 30-s intervals.

### Drug Treatment: Inhibiting GSK3 in the Flash of Light and Repeated Acoustic Startle Assay in Larvae

To test whether pharmacological inhibition of GSK-3 could reverse stress-reactivity phenotypes, 5 dpf zebrafish were exposed to BIO-acetoxime (HY-15356, Cambridge Bioscience), a potent and selective GSK-3 inhibitor. A concentration of 200 μM was used, corresponding to the highest tolerated dose shown not to impair larval locomotion [29].

The genotype of larvae used in this experiment (Fig. 4B) was known from the parental crosses, allowing allocation to treatment groups across 48-well plates. Wild-type larvae were obtained from *fzd9b*^+*/*+^  × *fzd9b*^+*/*+^ crosses, homozygous larvae from *fzd9b*^*−/−*^ × *fzd9b*^*−/−*^ crosses, and heterozygous larvae from *fzd9b*^+*/*+^  × *fzd9b*^*−/−*^ crosses. Individual larvae were not biopsied or genotyped before treatment. Each plate included 6 experimental conditions, with 4–6 replicates per condition. Condition placement within plates was randomised. Zebrafish were the result of either *fzd9b*^*−/−*^ or *fzd9b*^+*/*+^ in-crosses, or *fzd9b*^*−/−*^ x *fzd9b*^+*/*+^ crossings and reared under standardised conditions across multiple clutches and breeding tanks. Experiments were conducted over three consecutive days, using newly generated 5 dpf larvae on each day. Each genotype × treatment group included 24 larvae on day 1, 12 on day 2, and 6 on day 3, yielding a total of 42 larvae per group.

Fish from each genotype (*fzd9b*^+*/*+^, *fzd9b*^+/-^, *fzd9b*^*−/−*^) were individually housed and incubated for 90 min in the dark within a DanioVision Chamber under one of three conditions: water, DMSO (vehicle control), or BIO-acetoxime (200 μM). This resulted in nine treatment groups: 1) *fzd9b*^+*/*+^ water, 2) *fzd9b*^+*/*+^ DMSO, 3) *fzd9b*^+*/*+^ 200 μM BIO-acetoxime, 4) *fzd9b*^+/-^ water, 5) *fzd9b*^+/-^ DMSO, 6) *fzd9b*^+/-^ 200 μM BIO-acetoxime, 7) *fzd9b*^*−/−*^ water*)*, 8) *fzd9b*^*−/−*^ DMSO, 9) *fzd9b*^*−/−*^ 200 μM BIO-acetoxime.

#### Combined Light–Startle Behavioural Assay

Following drug exposure, larvae were tested using a behavioural paradigm combining a light–locomotor assay and an acoustic startle habituation protocol, adapted as previously described [28]. This combined protocol allowed all experimental groups to be tested within a single session (09:00–14:00), ensuring consistency across conditions. The test consisted of: 1-min baseline recording in darkness, 2-s flash of white light, 2-min post-light dark period, 25 consecutive acoustic/vibration stimuli (2-s interstimulus interval). Locomotor activity was recorded using EthoVision XT software (Noldus), and data were exported in 1-s bins for analysis.

### Statistics

Molecular data were analysed with GraphPad Prism 9.0.2 for Windows (GraphPad Software, San Diego, California USA, www.graphpad.com). Each dataset was visually assessed for normal distribution with the homoscedasticity and the normal QQ plots. If normality assumptions were violated, a non-parametric test was used. Data from qPCR analysis was analysed using a two-way repeated measures ANOVA with “genotype” and “time” as factors and “subject” as matched set and using Bonferroni’s multiple comparison test. Data from the % of tail *cmyb* + was analysed using the non-parametric Kruskal–Wallis test (unpaired samples) using Dunn’s test to correct for multiple comparisons. All effects are reported significant at *p* < 0.05. Data from the acetylated tubulin immunostaining experiments were analysed using unpaired two-tailed Student’s t-tests to compare genotypes within each experiment.

Data from all larval behavioural assays (5 dpf) and novel tank assay were analysed similarly as our previous publication [28]. Briefly, we used RStudio (version 1.2.5042) and results reported with respect to a type-1 error rate of α = 0.05. R packages used include: ‘lme4’ with, if applicable, a post-hoc Tukey test using the R package ‘multcomp’. Percentage of fish responding to stimulus was analysed with GraphPad Prism 9.0.2 using Anova with repeat measures to look at interaction between total number of responders in all TAP events and genotype as explanatory variables. When applicable, Tukey test was used to correct for multiple comparisons.

Data from the sociability assay was analysed using GraphPad Prism 9.0.2 for Windows similarly as previous described [28]. In short, a two-tailed Wilcoxon test to assess changes in SPI in each condition (genotype, contrast) and a Kruskal–Wallis test with repeated measures and applying Dunn’s multiple comparisons test for correlation index. Statistical significance was defined at *p* < 0.05.

## Supplementary Information

Below is the link to the electronic supplementary material.

Supplementary file1 (DOCX 5689 KB)

## Data Availability

Generated data can be found at https://zenodo.org/ (10.5281/zenodo.10548489).
