# Supplementary material for: Disrupting fzd9b in Zebrafish Recapitulates Stress- and Anxiety-Like Behaviours Relevant to Williams Syndrome
Source: Mol Neurobiol. 2026 Aug 24;63(1):853. doi: 10.1007/s12035-026-06127-w (PMC13503493; doi:10.1007/s12035-026-06127-w)
Supplement: Supplementary file 1 [file 12035_2026_6127_MOESM1_ESM.docx]

**Supplementary Figure 1. Characterization of the *fzd9b* 52bp deletion (*fzd9b^52bpDEL^*) line. A)** Resulting amino acids (aa) sequences blast for wildtype (WT, above) and mutants (below) of the portion of exon 1 used for genotyping (Supplementary Figure 1). Red circle indicates mutation starting site, changed aa are in red and stop codon marked as a dash (-). **B)** Relative change in gene expression (log_10_) assessed by qPCR between 5 dpf larvae from each genotype showing that in *fzd9b^+/-^* fish (heterozygous, HET) the expression of *fzd9b* is significantly increased with respect to *fzd9b^+/+^*. Figure shows individual values (N = 5 per genotype, each representing a group of 16 larvae combined) and mean ± standard error mean (SEM). **C)** Quantification of the percentage of ISH against *cmyb* at the tail of 40 hpf embryos from the three genotypes (N = 7); right panel shows representative embryos. **D)** Quantification of neuronal branch points following acetylated tubulin immunostaining and skeleton analysis in larval tails; graphs show individual values and mean ± SEM. **E)** qPCR analysis of the relative change in gene expression for *wnt5a*, *wnt5b*, *wnt9a*, *scn4aa*, *scn4ab* and *tafa5l* for pools of 5 dpf larvae (same as panel B) for the three genotypes. Graphs show box and whiskers with mean (central horizontal line) and minimum to maximum points. Dots represent biological replicates. In all cases: * *p* < 0.05, ** *p* < 0.01, *** *p* < 0.001, **** *p* < 0.0001, vs. corresponding pairwise comparison. Scale bars represent 25 µm.

**Supplementary Figure 2**. DNA blast for the portion of *fzd9b*’s exon 1 amplified by PCR used for genotyping. **A)** *fzd9b^22bpDEL^* line and **B)** *fzd9b^52bpDEL^* line. In both cases, the above sequence is for WT and below for mutants. Primers used are highlighted in yellow, crRNA site in blue, PAM site in grey and restriction enzyme over-lined.

**Supplementary Figure 3.** **A)** Functional domains and **B)** 3D conformation for the Fzd9b receptor and the two small truncated proteins produced in the mutant lines. In both lines, the resulting truncated proteins only contain the Cysteine-rich Wnt-binding domain (CRD) while the intramembrane portion and the Carboxy-Terminal Tail (CTT) are absent. CRD is located extracellularly and is needed for binding Wnt proteins. Note that, although both truncated proteins are similar, the isolectric point (pI) of the 52 bp deletion is close to physiological levels (pI = 8) while the 22 bp differs (pI = 5). Furthermore, the additional 44 aa preceding the premature stop codon for the 52 bp deletion (dark yellow) codes for cysteines or unknown (X) aa. Functional domains were assessed with NCBI’s CD-search tool (https://www.ncbi.nlm.nih.gov/Structure/cdd/wrpsb.cgi) and 3D structure with Phyre^2^ (http://www.sbg.bio.ic.ac.uk/~phyre2/html/page.cgi?id=index).

In both cases, the ANOVA statistical assay showed a significant difference in the expression of this mRNA by qPCR between genotypes (*fzd9b^22bpDEL^*: F (2, 9) = 15.95, p = 0.0011; *fzd9b^52bpDEL^*: F (2, 12) = 6.866, p = 0.0103), and post-hoc analysis revealed it to be driven by a significant increase in the heterozygous (fzd9b^+/-,^ HET) when compared to wildtype (fzd9b^+/+^, WT) siblings (HET vs. WT; *fzd9b^22bpDEL^*: p = 0.0021, Fig. 1C; *fzd9b^52bpDEL^*: p = 0.0033) and homozygous (fzd9b^-/-^, HOM; HET vs. HOM; *fzd9b^22bpDEL^*: p = 0.0005; *fzd9b^52bpDEL^*: p = 0.0370).

**Supplementary Figure 4. Disrupting *fzd9b* does not alter zebrafish responses in the forced light dark (FLD) assay.** Mean distance moved per genotype during the baseline and the three light-dark cycles (10 minutes each) for **A)** the *fzd9b^22bpDEL^* line (n_WT_ = 48, n_HET_ = 93, N_HOM_ = 47) and, **B)** the *fzd9b^52bpDEL^* line (n_WT_ = 49, n_HET_ = 91, n_HOM_ = 48). Data shows mean ± SEM.

**Supplementary Figure 5. Behavioural and molecular phenotypes in the *fzd9b^52bpDEL^* line.**

**A)** Proportion of responders to acoustic startle across all stimuli at 5 dpf in *fzd9b^+/+^* (n = 46), *fzd9b^+/-^* (n = 85), and *fzd9b^-/-^* (n = 47) larvae; graph shows mean ± SEM. **B)** Relative gene expression (log₁₀ scale) of HPI axis genes: *mr*, *gr*, and *crhb*, measured by qPCR in pooled 5 dpf larvae (n = 5 per genotype; 16 larvae per pool); points represent biological replicates; bars show mean ± SEM. **C)** Correlation index (r) of social preference in juvenile zebrafish (20–22 dpf) from the *fzd9b^52bpDEL^* line, comparing all tested contrasts (0 vs. 0, vs. 0 vs. 1, and vs. 0 vs. 3) for *fzd9b^+/+^* (dark grey), *fzd9b^+/-^* (light grey), and *fzd9b^-/-^* (white); each dot represents an individual; bars show mean ± SEM. In all cases: * p < 0.05, *** p < 0.001 vs. corresponding pairwise comparison.

**Supplementary Figure 6. Novel tank diving assay shows mild differences in anxiety-like behaviours in the *fzd9b^52bpDEL^* line. A)** Distance travelled, **B)** distance to the bottom, **C)** Proportion of time in the bottom third and **D)** top halve visits for the three genotypes (*fzd9b^+/+^*, *fzd9b^+/-^*, *fzd9b^-/-^*) in 30 seconds time bins. Although there was no difference by genotype in the distance to the bottom, the proportion of time in the bottom third for the *fzd9b^+/-^* was significantly different than the other genotypes and *fzd9b^+/-^* also visited significantly more the top half part of the tank. Graphs show mean ± SEM. Graph B includes the n number. * p < 0.0 5 vs. 8^th^ time point until the last, from same genotype (HET); & p < 0.0 5 vs. 4^th^ time point until the last, from same genotype (HET); # p < 0.0 5 vs. 4^th^ time point from same genotype (WT).

**Supplementary Figure 7. Wnt pathway and fzds expression, and behavioural response to BIO-acetoxime in 5 dpf zebrafish from the *fzd9b^52bpDEL^* line. A)** Relative gene expression (log₁₀ scale) of Wnt/β-catenin pathway genes (*cyclind1*, *myca*, *ctnnb1*, *ctnnb2*, *axin2*, *sfrp2*) measured by qPCR in pooled 5 dpf larvae (N = 5 per genotype; 16 larvae per pool). Graphs show individual values and mean ± SEM. **B)** Relative gene expression (log₁₀ scale) of other frizzled receptor genes (*fzd7a*, *fzd7b*, *fzd8a*, *fzd8b*, *fzd9a*, *fzd10*) measured by qPCR in pooled 5 dpf larvae (N = 5 per genotype; 16 larvae per pool). Graphs show individual values and mean ± SEM. **C)** Proportion of responders across stimuli for each treatment condition; each genotype × treatment group included n = 42 larvae; data represent mean ± SEM. * *p* < 0.05, ** *p* < 0.01 vs. corresponding pairwise comparison.

**Supplementary Figure 8. A)** Protein alignment of human FZD9 with zebrafish Fzd9b and Fzd9a using COBALT (NCBI Multiple Sequence Alignment Viewer, v1.25.1). Red uppercase letters indicate conserved residues; blue indicate mismatches; lowercase letters mark non-aligning positions. **B)** Column conservation scores based on residue entropy: red indicates high conservation, blue indicates low. Functional domains are highlighted. **C)** Ribbon plot of *fzd9b* and *fzd9a* expression in wild-type zebrafish from ZFIN (https://zfin.org/), compiled from published and submitted sources.

**Supplementary Table 1. Changes in gene expression.** Table shows the expression change for *fzd9b* downstream interacting genes, genes associated with the HPI axis, genes from the Wnt-signalling pathway, and frizzled receptors for both mutant lines, *fzd9b^22bpDEL^* (left) and *fzd9b^52bpDEL^* (right). Table shows mean difference (Mean Diff.), 95% Confidence Interval of the difference (95% CI of diff.), adjusted P value and significance summary for each contrast/gene studied. * < 0.05, ** < 0.01, *** < 0.001, ns > 0.05 vs. corresponding contrast.

|  |  | ***fzd9b^22bpDEL^*** | | | | ***fzd9b^52bpDEL^*** | | | |  |
| --- | --- | --- | --- | --- | --- | --- | --- | --- | --- | --- |
|  | *gene* / contrast | Mean Diff. | 95% CI of diff. | Summary | Adjusted P Value | Mean Diff. | 95% CI of diff. | Summary | Adjusted P Value |  |
| *fzd9b* interacting genes | *wnt5a* |  |  |  |  |  |  |  |  |  |
|  | *fzd9b*^+/+^ vs.*fzd9b*^+/-^ | -0.8984 | -2.872 to 1.075 | ns | 0.7969 | -1.85 | -3.966 to 0.2662 | ns | 0.1065 |  |
|  | *fzd9b*^+/+^ vs.*fzd9b-*^/-^ | 0.05654 | -1.917 to 2.030 | ns | >0.9999 | -0.2132 | -2.329 to 1.903 | ns | >0.9999 |  |
|  | *fzd9b*^+/-^ vs.*fzd9b-*^/-^ | 0.9549 | -1.019 to 2.928 | ns | 0.7112 | 1.637 | -0.4794 to 3.753 | ns | 0.1859 |  |
|  | *wnt5b* |  |  |  |  |  |  |  |  |  |
|  | *fzd9b*^+/+^ vs.*fzd9b*^+/-^ | -1.882 | -3.856 to 0.09128 | ns | 0.0663 | -2.194 | -4.310 to -0.07769 | * | 0.0396 |  |
|  | *fzd9b*^+/+^ vs.*fzd9b-*^/-^ | -2.199 | -4.172 to -0.2251 | * | 0.0241 | -0.846 | -2.962 to 1.270 | ns | 0.9913 |  |
|  | *fzd9b*^+/-^ vs.*fzd9b-*^/-^ | -0.3164 | -2.290 to 1.657 | ns | >0.9999 | 1.348 | -0.7683 to 3.464 | ns | 0.3685 |  |
|  | *wnt9a* |  |  |  |  |  |  |  |  |  |
|  | *fzd9b*^+/+^ vs.*fzd9b*^+/-^ | -0.3392 | -2.313 to 1.634 | ns | >0.9999 | -0.8257 | -2.942 to 1.291 | ns | >0.9999 |  |
|  | *fzd9b*^+/+^ vs.*fzd9b-*^/-^ | -1.61 | -3.583 to 0.3636 | ns | 0.1465 | 0.2021 | -1.914 to 2.318 | ns | >0.9999 |  |
|  | *fzd9b*^+/-^ vs.*fzd9b-*^/-^ | -1.271 | -3.244 to 0.7028 | ns | 0.3524 | 1.028 | -1.089 to 3.144 | ns | 0.7134 |  |
|  | *scn4aa* |  |  |  |  |  |  |  |  |  |
|  | *fzd9b*^+/+^ vs.*fzd9b*^+/-^ | -0.4108 | -2.384 to 1.563 | ns | >0.9999 | -1.692 | -3.808 to 0.4241 | ns | 0.1616 |  |
|  | *fzd9b*^+/+^ vs.*fzd9b-*^/-^ | -1.227 | -3.201 to 0.7460 | ns | 0.3905 | 0.9494 | -1.167 to 3.066 | ns | 0.8255 |  |
|  | *fzd9b*^+/-^ vs.*fzd9b-*^/-^ | -0.8167 | -2.790 to 1.157 | ns | 0.9333 | 2.642 | 0.5253 to 4.758 | ** | 0.0093 |  |
|  | *scn4ab* |  |  |  |  |  |  |  |  |  |
|  | *fzd9b*^+/+^ vs.*fzd9b*^+/-^ | -0.8412 | -2.815 to 1.132 | ns | 0.8907 | -2.364 | -4.480 to -0.2477 | * | 0.0233 |  |
|  | *fzd9b*^+/+^ vs.*fzd9b-*^/-^ | -1.599 | -3.572 to 0.3745 | ns | 0.151 | -0.3057 | -2.422 to 1.811 | ns | >0.9999 |  |
|  | *fzd9b*^+/-^ vs.*fzd9b-*^/-^ | -0.7577 | -2.731 to 1.216 | ns | >0.9999 | 2.058 | -0.05795 to 4.175 | ns | 0.0593 |  |
|  | *tafa5l* |  |  |  |  |  |  |  |  |  |
|  | *fzd9b*^+/+^ vs.*fzd9b*^+/-^ | 1.067 | -0.9068 to 3.040 | ns | 0.562 | -2.906 | -5.022 to -0.7896 | ** | 0.0037 |  |
|  | *fzd9b*^+/+^ vs.*fzd9b-*^/-^ | -2.321 | -4.295 to -0.3477 | * | 0.0159 | -1.535 | -3.651 to 0.5816 | ns | 0.2391 |  |
|  | *fzd9b*^+/-^ vs.*fzd9b-*^/-^ | -3.388 | -5.361 to -1.414 | *** | 0.0003 | 1.371 | -0.7451 to 3.487 | ns | 0.3499 |  |
|  |  |  |  |  |  |  |  |  |  |  |
| HPI axis | *mr* |  |  |  |  |  |  |  |  |  |
|  | *fzd9b*^+/+^ vs.*fzd9b*^+/-^ | -0.8832 | -1.530 to -0.2368 | ** | 0.0051 | -0.1105 | -0.4948 to 0.2738 | ns | 0.9653 |  |
|  | *fzd9b*^+/+^ vs.*fzd9b^-^*^/-^ | -0.9177 | -1.621 to -0.2139 | ** | 0.0076 | -0.162 | -0.5462 to 0.2223 | ns | 0.8198 |  |
|  | *fzd9b*^+/-^ vs.*fzd9b^-^*^/-^ | -0.03449 | -0.7705 to 0.7015 | ns | >0.9999 | -0.05148 | -0.3972 to 0.2942 | ns | 0.976 |  |
|  | *gr* |  |  |  |  |  |  |  |  |  |
|  | *fzd9b*^+/+^ vs.*fzd9b*^+/-^ | -0.6182 | -1.265 to 0.02825 | ns | 0.0645 | -0.2887 | -0.6730 to 0.09560 | ns | 0.2349 |  |
|  | *fzd9b*^+/+^ vs.*fzd9b^-^*^/-^ | -0.452 | -1.156 to 0.2518 | ns | 0.3382 | -0.04154 | -0.4258 to 0.3428 | ns | 0.9998 |  |
|  | *fzd9b*^+/-^ vs.*fzd9b^-^*^/-^ | 0.1662 | -0.5698 to 0.9022 | ns | >0.9999 | 0.2471 | -0.09854 to 0.5928 | ns | 0.226 |  |
|  | *crhb* |  |  |  |  |  |  |  |  |  |
|  | *fzd9b*^+/+^ vs.*fzd9b*^+/-^ | -1.067 | -1.714 to -0.4207 | *** | 0.0008 | -0.3998 | -0.7841 to -0.01548 | * | 0.0378 |  |
|  | *fzd9b*^+/+^ vs.*fzd9b^-^*^/-^ | -0.9644 | -1.668 to -0.2607 | ** | 0.0049 | -0.3802 | -0,7645 to 0.004091 | ns | 0.0538 |  |
|  | *fzd9b*^+/-^ vs.*fzd9b^-^*^/-^ | 0.1027 | -0.6333 to 0.8387 | ns | >0.9999 | 0.01957 | -0.3261 to 0.3653 | ns | 0.9986 |  |
|  |  |  |  |  |  |  |  |  |  |  |
| Wnt pathway genes | *cyclind1* |  | | | |  | | | |  |
|  | *fzd9b*^+/+^ vs.*fzd9b*^+/-^ | -0.2321 | -1.282 to 0.8178 | ns | >0.9999 | -1.865 | -3.585 to -0.1458 | * | 0.0289 |  |
|  | *fzd9b*^+/+^ vs.*fzd9b^-^*^/-^ | 0.4303 | -0.7127 to 1.573 | ns | >0.9999 | -1.608 | -3.328 to 0.1113 | ns | 0.0744 |  |
|  | *fzd9b*^+/-^ vs.*fzd9b^-^*^/-^ | 0.6624 | -0.5330 to 1.858 | ns | 0.5298 | 0.2571 | -1.462 to 1.977 | ns | >0.9999 |  |
|  | *myca* |  | | | |  | | | |  |
|  |  |  |  |  |  |  |  |  |  |  |
|  | *fzd9b*^+/+^ vs.*fzd9b*^+/-^ | -0.2429 | -1.293 to 0.8070 | ns | >0.9999 | -1.018 | -2.738 to 0.7012 | ns | 0.4529 |  |
|  |  |  |  |  |  |  |  |  |  |  |
|  | *fzd9b*^+/+^ vs.*fzd9b^-^*^/-^ | -0.6157 | -1.759 to 0.5273 | ns | 0.5664 | -0.5126 | -2.232 to 1.207 | ns | >0.9999 |  |
|  | *fzd9b*^+/-^ vs.*fzd9b^-^*^/-^ | -0.3728 | -1.568 to 0.8226 | ns | >0.9999 | 0.5056 | -1.214 to 2.225 | ns | >0.9999 |  |
|  | *sfrp2* |  | | | |  | | | |  |
|  |  |  |  |  |  |  |  |  |  |  |
|  | *fzd9b*^+/+^ vs.*fzd9b*^+/-^ | -0.3685 | -1.418 to 0.6814 | ns | >0.9999 | -2.571 | -4.290 to -0.8512 | ** | 0.0014 |  |
|  | *fzd9b*^+/+^ vs.*fzd9b^-^*^/-^ | -1.742 | -2.885 to -0.5987 | ** | 0.0012 | -0.8797 | -2.599 to 0.8398 | ns | 0.6417 |  |
|  | *fzd9b*^+/-^ vs.*fzd9b^-^*^/-^ | -1.373 | -2.569 to -0.1779 | * | 0.0191 | 1.691 | -0.02848 to 3.410 | ns | 0.0554 |  |
|  | *ctnnb1* |  | | | |  | | | |  |
|  | *fzd9b*^+/+^ vs.*fzd9b*^+/-^ | -1.037 | -2.087 to 0.01245 | ns | 0.0538 | -0.7258 | -2.445 to 0.9937 | ns | 0.9129 |  |
|  | *fzd9b*^+/+^ vs.*fzd9b^-^*^/-^ | -1.23 | -2.373 to -0.08656 | * | 0.031 | -0.7853 | -2.505 to 0.9342 | ns | 0.8 |  |
|  | *fzd9b*^+/-^ vs.*fzd9b^-^*^/-^ | -0.1921 | -1.387 to 1.003 | ns | >0.9999 | -0.05951 | -1.779 to 1.660 | ns | >0.9999 |  |
|  | *ctnnb2* |  | | | |  | | | |  |
|  | *fzd9b*^+/+^ vs.*fzd9b*^+/-^ | 1.482 | 0.4319 to 2.532 | ** | 0.0029 | -0.6598 | -2.379 to 1.060 | ns | >0.9999 |  |
|  | *fzd9b*^+/+^ vs.*fzd9b^-^*^/-^ | 0.8639 | -0.2791 to 2.007 | ns | 0.2018 | -0.3111 | -2.031 to 1.408 | ns | >0.9999 |  |
|  | *fzd9b*^+/-^ vs.*fzd9b^-^*^/-^ | -0.6179 | -1.813 to 0.5775 | ns | 0.621 | 0.3487 | -1.371 to 2.068 | ns | >0.9999 |  |
|  | *axin2* |  | | | |  | | | |  |
|  | *fzd9b*^+/+^ vs.*fzd9b*^+/-^ | -1.126 | -2.176 to -0.07634 | * | 0.0316 | -0.5637 | -2.283 to 1.156 | ns | >0.9999 |  |
|  | *fzd9b*^+/+^ vs.*fzd9b^-^*^/-^ | -1.286 | -2.429 to -0.1427 | * | 0.0224 | -1.186 | -2.905 to 0.5335 | ns | 0.2857 |  |
|  | *fzd9b*^+/-^ vs.*fzd9b^-^*^/-^ | -0.1595 | -1.355 to 1.036 | ns | >0.9999 | -0.6223 | -2.342 to 1.097 | ns | >0.9999 |  |
|  |  |  |  |  |  |  |  |  |  |  |
| Fzd receptor genes | *fzd7a* |  | | | |  | | | |  |
|  | *fzd9b*^+/+^ vs.*fzd9b*^+/-^ | -0.6009 | -1.670 to 0.4679 | ns | 0.5114 | -1.144 | -3.172 to 0.8849 | ns | 0.5138 |  |
|  | *fzd9b*^+/+^ vs.*fzd9b^-^*^/-^ | -1.175 | -2.338 to -0.01136 | * | 0.0471 | -1.738 | -3.767 to 0.2907 | ns | 0.1177 |  |
|  | *fzd9b*^+/-^ vs.*fzd9b^-^*^/-^ | -0.574 | -1.791 to 0.6429 | ns | 0.7469 | -0.5943 | -2.623 to 1.434 | ns | >0.9999 |  |
|  | *fzd7b* |  | | | |  | | | |  |
|  |  |  |  |  |  |  |  |  |  |  |
|  | *fzd9b*^+/+^ vs.*fzd9b*^+/-^ | 0.2556 | -0.8132 to 1.324 | ns | >0.9999 | -1.805 | -3.834 to 0.2235 | ns | 0.0973 |  |
|  |  |  |  |  |  |  |  |  |  |  |
|  | *fzd9b*^+/+^ vs.*fzd9b^-^*^/-^ | 0.2172 | -0.9464 to 1.381 | ns | >0.9999 | -1.49 | -3.519 to 0.5384 | ns | 0.2279 |  |
|  | *fzd9b*^+/-^ vs.*fzd9b^-^*^/-^ | -0.03843 | -1.255 to 1.178 | ns | >0.9999 | 0.315 | -1.714 to 2.344 | ns | >0.9999 |  |
|  | *fzd8a* |  | | | |  | | | |  |
|  |  |  |  |  |  |  |  |  |  |  |
|  | *fzd9b*^+/+^ vs.*fzd9b*^+/-^ | -1.561 | -2.630 to -0.4926 | ** | 0.002 | -0.5179 | -2.547 to 1.511 | ns | >0.9999 |  |
|  | *fzd9b*^+/+^ vs.*fzd9b^-^*^/-^ | -1.816 | -2.979 to -0.6524 | *** | 0.0009 | -0.715 | -2.744 to 1.314 | ns | >0.9999 |  |
|  | *fzd9b*^+/-^ vs.*fzd9b^-^*^/-^ | -0.2545 | -1.471 to 0.9624 | ns | >0.9999 | -0.1971 | -2.226 to 1.832 | ns | >0.9999 |  |
|  | *fzd8b* |  | | | |  | | | |  |
|  | *fzd9b*^+/+^ vs.*fzd9b*^+/-^ | -1.802 | -2.871 to -0.7334 | *** | 0.0003 | -2.302 | -4.331 to -0.2732 | * | 0.0207 |  |
|  | *fzd9b*^+/+^ vs.*fzd9b^-^*^/-^ | -0.6361 | -1.800 to 0.5275 | ns | 0.5472 | -1.728 | -3.757 to 0.3003 | ns | 0.1209 |  |
|  | *fzd9b*^+/-^ vs.*fzd9b^-^*^/-^ | 1.166 | -0.05074 to 2.383 | ns | 0.0645 | 0.5735 | -1.455 to 2.602 | ns | >0.9999 |  |
|  | *fzd9a* |  | | | |  | | | |  |
|  | *fzd9b*^+/+^ vs.*fzd9b*^+/-^ | 1.223 | 0.1540 to 2.292 | * | 0.0197 | -2.166 | -4.195 to -0.1375 | * | 0.0324 |  |
|  | *fzd9b*^+/+^ vs.*fzd9b^-^*^/-^ | 1.409 | 0.2455 to 2.573 | * | 0.0125 | -1.389 | -3.418 to 0.6393 | ns | 0.2926 |  |
|  | *fzd9b*^+/-^ vs.*fzd9b^-^*^/-^ | 0.1862 | -1.031 to 1.403 | ns | >0.9999 | 0.7768 | -1.252 to 2.805 | ns | >0.9999 |  |
|  | *fzd10* |  | | | |  | | | |  |
|  | *fzd9b*^+/+^ vs.*fzd9b*^+/-^ | 0.5723 | -0.4965 to 1.641 | ns | 0.5743 | -1.505 | -3.533 to 0.5241 | ns | 0.2197 |  |
|  | *fzd9b*^+/+^ vs.*fzd9b^-^*^/-^ | 0.2934 | -0.8701 to 1.457 | ns | >0.9999 | -1.259 | -3.288 to 0.7698 | ns | 0.3979 |  |
|  | *fzd9b*^+/-^ vs.*fzd9b^-^*^/-^ | -0.2788 | -1.496 to 0.9380 | ns | >0.9999 | 0.2457 | -1.783 to 2.274 | ns | >0.9999 |  |

**Supplementary Table 2.** Details of statistical analysis for the forced light dark transition assay performed in 5 days post fertilization larvae (WT, HET, HOM) for both *fzd9b^22bpDEL^* and *fzd9b^52bpDEL^* (Experiment 2).

| Line | Dependent variable | Period of the assay | Main effect | p value | χ2 | Degrees of freedom |
| --- | --- | --- | --- | --- | --- | --- |
| *fzd9b^22bpDEL^* | Distance travelled | Baseline | Time | p < 0.05 | 16.379 | 8 |
| *fzd9b^22bpDEL^* | Distance travelled | Light 1 (min 10-20) | Time | p < 0.0001 | 32.4988 | 8 |
| *fzd9b^22bpDEL^* | Distance travelled | Light 2 (min 30-40) | Time | p < 0.0001 | 97.694 | 8 |
| *fzd9b^22bpDEL^* | Distance travelled | Light 3 (min 50-60) | Time | p < 0.0001 | 45.4471 | 8 |
| *fzd9b^22bpDEL^* | Distance travelled | Dark 1 (min 20-30) | Time | p < 0.0001 | 81.2740 | 8 |
| *fzd9b^22bpDEL^* | Distance travelled | Dark 2 (min 40-50) | Time | p < 0.0001 | 61.8538 | 8 |
| *fzd9b^22bpDEL^* | Distance travelled | Dark 3 (min 60-70) | Time | p < 0.0001 | 63.6949 | 8 |
| *fzd9b^22bpDEL^* | Slope of recovery | Light periods | Time | p < 0.05 | 554.916 | 29 |
| *fzd9b^52bpDEL^* | Distance travelled | Baseline | Time | p < 0.0001 | 38.9689 | 8 |
| *fzd9b^52bpDEL^* | Distance travelled | Light 1 (min 10-20) | Time | p < 0.0001 | 67.1889 | 8 |
| *fzd9b^52bpDEL^* | Distance travelled | Light 2 (min 30-40) | Time | p < 0.0001 | 101.8495 | 8 |
| *fzd9b^52bpDEL^* | Distance travelled | Light 3 (min 50-60) | Time | p < 0.0001 | 129..7344 | 8 |
| *fzd9b^52bpDEL^* | Distance travelled | Dark 1 (min 20-30) | Time | p < 0.0001 | 115.1714 | 8 |
| *fzd9b^52bpDEL^* | Distance travelled | Dark 2 (min 40-50) | Time | p < 0.0001 | 92.7437 | 8 |
| *fzd9b^52bpDEL^* | Distance travelled | Dark 3 (min 60-70) | Time | p < 0.0001 | 114.3604 | 8 |
| *fzd9b^52bpDEL^* | Slope of recovery | Light periods | Time | p < 0.05 | 114.3604 | 29 |

**Supplementary Table 3.** Details of statistical analysis for habituation and response to acoustic startle assay performed in 5 days post fertilization larvae (WT, HET, HOM) for both *fzd9b^22bpDEL^* and *fzd9b^52bpDEL^* in presence of the selective GSK-3 inhibitor BIO-acetoxime (200 µM). Baseline 1 refers to the basal locomotion before the flash of light, whereas baseline 2 refers to the basal locomotion after the flash of light and before the acoustic stimuli.

| Line | Condition | Dependent variable | Period of the assay | Main effect | p value | χ2 | Degrees of freedom |
| --- | --- | --- | --- | --- | --- | --- | --- |
| *fzd9b^22bpDEL^* | DMSO | Distance travelled | Baseline 1 | Time | p < 0.05 | 65.3706 | 59 |
| *fzd9b^22bpDEL^* | DMSO | Distance travelled | Baseline 2 | Time | p < 2e-16 | 7354.1204 | 119 |
| *fzd9b^22bpDEL^* | DMSO | Magnitude of response | Light flash | Time | p < 2e-16 | 115.773 | 1 |
| *fzd9b^22bpDEL^* | DMSO | Slope of recovery | Light flash | Time | p < 0.05 | 6.1717 | 1 |
| *fzd9b^22bpDEL^* | DMSO | Distance travelled | Taps | Genotype | p < 0.05 | 7.3771 | 2 |
| *fzd9b^22bpDEL^* | DMSO | Distance travelled | Taps | Genotype * Time | p < 2e-16 | 148.0517 | 48 |
| *fzd9b^22bpDEL^* | DMSO | Proportion of responders | Taps | - | p > 0.05 | - | - |
| *fzd9b^22bpDEL^* | DMSO | Rate of habituation | Taps | Genotype | p < 3.972e-13 | 57.109 | 2 |
| *fzd9b^22bpDEL^* | DMSO | Rate of habituation | Taps | Genotype * Stimulus number | p < 2.2e-16 | 554.916 | 27 |
| *fzd9b^22bpDEL^* | BIO-acetoxime 200µM | Distance travelled | Baseline 1 | Time | p < 0.05 | 53.4153 | 59 |
| *fzd9b^22bpDEL^* | BIO-acetoxime 200µM | Distance travelled | Baseline 2 | Time | p < 2e-16 | 29333.0044 | 119 |
| *fzd9b^22bpDEL^* | BIO-acetoxime 200µM | Magnitude of response | Light flash | Time | p < 2e-16 | 127.4317 | 1 |
| *fzd9b^22bpDEL^* | BIO-acetoxime 200µM | Slope of recovery | Light flash | Time | p < 0.001 | 2.0533 | 1 |
| *fzd9b^22bpDEL^* | BIO-acetoxime 200µM | Distance travelled | Taps | Genotype * Drug * Time | p < 0.05 | 71.5187 | 48 |
| *fzd9b^22bpDEL^* | BIO-acetoxime 200µM | Proportion of responders | Taps | - | p > 0.05 | - | - |
| *fzd9b^22bpDEL^* | BIO-acetoxime 200µM | Rate of habituation | Taps | Genotype * Drug | p < 7.653e-9 | 37.847 | 4 |
| *fzd9b^22bpDEL^* | BIO-acetoxime 200µM | Rate of habituation | Taps | Genotype * Drug * Stimulus number | p < 2.2e-16 | 1076.527 | 29 |
| *fzd9b^52bpDEL^* | DMSO | Distance travelled | Baseline 1 | Time | p < 0.01 | 65.3706 | 59 |
| *fzd9b^52bpDEL^* | DMSO | Distance travelled | Baseline 2 | Time | p < 2e-16 | 7354.1204 | 119 |
| *fzd9b^52bpDEL^* | DMSO | Magnitude of response | Light flash | Time | p < 2e-16 | 115.773 | 1 |
| *fzd9b^52bpDEL^* | DMSO | Slope of recovery | Light flash | Time | p < 0.05 | 6.1717 | 1 |
| *fzd9b^52bpDEL^* | DMSO | Distance travelled | Taps | Time | p < 2e-16 | 686.671 | 24 |
| *fzd9b^52bpDEL^* | DMSO | Proportion of responders | Taps | - | p < 0.05 | - | - |
| *fzd9b^52bpDEL^* | DMSO | Rate of habituation | Taps | Genotype | p < 2e-16 | 338.3 | 2 |
| *fzd9b^52bpDEL^* | DMSO | Rate of habituation | Taps | Genotype * Time | p < 2e-16 | 1008.4 | 27 |
| *fzd9b^52bpDEL^* | BIO-acetoxime 200µM | Distance travelled | Baseline 1 | Time | p < 0.05 | 53.4153 | 59 |
| *fzd9b^52bpDEL^* | BIO-acetoxime 200µM | Distance travelled | Baseline 2 | Time | p < 2e-16 | 12819.9488 | 119 |
| *fzd9b^52bpDEL^* | BIO-acetoxime 200µM | Magnitude of response | Light flash | Time | p < 2e-16 | 217.8463 | 1 |
| *fzd9b^52bpDEL^* | BIO-acetoxime 200µM | Slope of recovery | Light flash | Time | p < 0.05 | 7.4217 | 1 |
| *fzd9b^52bpDEL^* | BIO-acetoxime 200µM | Distance travelled | Taps | Time | p < 2e-16 | 1332.3594 | 24 |
| *fzd9b^52bpDEL^* | BIO-acetoxime 200µM | Proportion of responders | Taps | - | p > 0.05 | - | - |
| *fzd9b^52bpDEL^* | BIO-acetoxime 200µM | Rate of habituation | Taps | Drug | p < 9.555e-9 | 37.414 | 4 |
| *fzd9b^52bpDEL^* | BIO-acetoxime 200µM | Rate of habituation | Taps | Genotype * Drug * Stimulus number | p < 2e-16 | 893.929 | 29 |

**Supplementary Table 4.** Genes and corresponding primers used for qPCR.

| Gene name | ID number | forward (5'-3') | reverse (5'-3') | Length (bp) | Efficiency (%) | Source |
| --- | --- | --- | --- | --- | --- | --- |
| *fzd9b* | ENSDARG00000014673 | TCCAGAATGGGAAGCTCACCTC | TCTCTCCAGGTCATAAGAGCCG | 99 | 100 | Own produced |
| *scn4aa* | ENSDART00000170961.2 | CGGCGATGTTTTGCATCAGTC | ACACGCAATTGGACAGGATGG | 114 | 99.1 | Own produced |
| *scn4ab* | ENSDART00000044157.8 | TGTTCATGGCGATGAGCGAC | GCAGAAACCTCTGGACAGAACC | 110 | 82 | Own produced |
| *tafa5l* | ENSDART00000133348.2 | TCAACCCAAGCGCACTATCG | CACACCACTGCTTGCTTTTCAC | 128 | 89 | Own produced |
| *wnt5a* | ENSDART00000164739.2 | AAAGACACTCCACACACACTGC | AGAGGATTCATCGCCAGAGACC | 107 | 88.6 | Own produced |
| *wnt5b* | ENSDART00000186829.1 | AGGACACCTACTTCTGGCAGTG | GGGGTTCATGGCTAATGACCAC | 91 | 97 | Own produced |
| *wnt9a* | ENSDART00000130349.3 | TTGAGAGATGGAACTGCACGC | GTGAGGCCCGCTGAAGAAATAG | 106 | 92.3 | Own produced |
| *crhb* (HPI axis) | NM_001007379.1 | CGAGACATCCCAGTATCCAAAAAG | TCCAACAGACGCTGCGTTAA | 60 | 111 | Doi: 10.1038/s41598-020-79615-1 |
| *mr* (HPI axis) | NM_001100403 | CTTCCAGGTTTCCGCAGTCTAC | GGAGGAGAGACACATCCAGGAAT | 75 | 105 | Doi: 10.1038/s41598-020-79615-1 |
| *gr* (HPI axis) | NM_001020711.3 | ACTCCATGCACGACTTGGTG | GCATTTCGGGAAACTCCACG | 90 | 92 | Doi: 10.1038/s41598-020-79615-1 |
| *axin2* | NM_131561.1 | GGACACTTCAAGGAACAACTAC | CCTCATACATTGGCAGAACTG | 133 | 110.4 | Own produced |
| *srfp2* | NM_001077384.3 | ACCAGTCACTAAGGAAGTTCCAA | GGCTTTTCCTTGCAGGCATC | 51 | 115 | Own produced |
| *cdx1a* | NM_212836.3 | GGCATGCACTGCTTAAAACATATG | TCAGAACTCGGAGGATTATTTT | 96 | 107 | Own produced |
| *myca* | NM_131412.1 | TGACTGTGGAAAAGCGACAG | GCTGCTGTTGATGCTGTGAT | 197 | 109 | Own produced |
| *ctnnb1* | NM_131059.2 | GACAGGACGACCCAAGCTAC | GCCGTCTACGGGGTAATCAG | 128 | 110 | Own produced |
| *ctnnb2* | NM_001001889.1 | ACGCTCAGGATCTGATGGAC | AGGCACTTTCTGAACCTCCA | 164 | 102 | Own produced |
| *ccnd1* | NM_131025.4 | GCCAAACTGCCTATACATCAG | TGTCGGTGCTTTTCAGGTAC | 169 | 85 | Own produced |
| *fzd7a* | NM_131139.2 | ACATGCAGACGTGCAAGAGA | AACAGTCCGCACGAGACATT | 271 | 81.05 | Own produced |
| *fzd7b* | NM_170763.3 | ATTCAACAGGAGTGGCGGAG | ACTCGTACAACAAGGCGAGG | 197 | 104.66 | Own produced |
| *fzd8a* | NM_130918.2 | CAAGTGAAGCGGAGATGGGT | CTGGTTGGGCATGTAGGTGT | 231 | 113.6 | Own produced |
| *fzd8b* | NM_131553.1 | AAACCGGACTACGCTGTGTT | GAGCTTGCGTCGCTGTAAAC | 182 | 121.53 | Own produced |
| *fzd9a* | XM_021476637.3 | GACCAGCCATAAGCCCTGTT | AAGGCTTATAAACGGCCCCC | 231 | 112.43 | Own produced |
| *fzd10* | NM_130917.2 | GAAGGTAGCAGGAGACGAGC | CACGCCGATCCTAACCATCA | 220 | 98.03 | Own produced |
| *ef1a* (housekeeping) | ENSDARG00000020850 | CTGGAGGCCAGCTCAAACAT | ATCAAGAAGAGTAGTACCGCTAGCATTAC | 87 | 88.4 | Doi:10.3389/fnbeh.2015,00271 |
| *rpl13α* (housekeeping) | ENSDARG00000044093 | TCTGGAGGACTGTAAGAGGTATGC | AGACGCACAATCTTGAGAGCAG | 148 | 80 | Doi:10.3389/fnbeh.2015,00271 |
| *actb2* (housekeeping) | NM_181601.5 | CGAGCTGTCTTCCCATCCA | TCACCAACGTAGCTGTCTTTCTG | 86 | 95 | Doi: 10.1038/s41598-020-79615-1 |
